# The activation of complement C5a-C5aR1 axis in astrocytes facilitates the neuropathogenesis due to EV-A71 infection by upregulating CXCL1

**DOI:** 10.1128/jvi.01514-24

**Published:** 2024-12-16

**Authors:** Peiyu Zhu, Wangquan Ji, Dong Li, Fang Wang, Tiantian Sun, Haiyan Yang, Shuaiyin Chen, Weiguo Zhang, Yuefei Jin, Guangcai Duan

**Affiliations:** 1 Department of Epidemiology, College of Public Health, Zhengzhou University 12636 , Zhengzhou, Henan, China; 2 Department of Infectious Diseases, Children’s Hospital Affiliated to Zhengzhou University, Henan Children's Hospital, Zhengzhou Children's Hospital 595848 , Zhengzhou, Henan, China; 3 Suzhou Institute of Systems Medicine, Chinese Academy of Medical Sciences 570570 , Suzhou, China; St. Jude Children's Research Hospital, Memphis, Tennessee, USA

**Keywords:** Enterovirus A71, C5a, C5aR1, astrocytes, neuropathogenesis

## Abstract

Enterovirus A71 (EV-A71) is a common small RNA virus that is highly neuroinvasive. Emerging evidence indicates that the complement fragment C5a and its receptor C5aR1 are important drivers of neuroinflammation. However, the potential role of the C5a–C5aR1 axis in EV-A71 encephalitis remains largely elusive. Our previous studies revealed that EV-A71 can infect astrocytes and result in complement activation *in vivo*. Here, we investigated how complement factors interact with astrocytes to promote a severe inflammatory response upon EV-A71 infection. Our data revealed that EV-A71 infected mainly astrocytes and caused astrocyte activation in the mouse brain, which was further verified in patients with EV-A71 infection and U87-MG cells. Notably, EV-A71 infection led to activation of the C5a–C5aR1 axis in U87-MG cells, and knockdown (siC5aR1) or blockade (PMX53) of C5aR1 significantly suppressed EV-A71-induced astrocyte activation and proinflammatory cytokine (e.g., CXCL1) production. Next, the activation of the C5a–C5aR1 axis in mouse astrocytes was confirmed. Compared with C5aR1 knockout mice, wild-type mice presented more severe symptoms and lower survival rates after EV-A71 infection. C5aR1 deficiency or blockade significantly reduced EV-A71-induced pathological damage and proinflammatory cytokine production in the mouse brain. Importantly, an increased level of soluble C5a was strongly correlated with the severity of symptoms in patients with EV-A71 infection. By using confocal microscopy, primary astrocytes, and human specimens, we observed that the increase in CXCL1 levels resulted mainly from astrocytes. Neutralizing CXCL1 significantly alleviated the neuropathological changes caused by EV-A71 infection, and the production of CXCL1 in astrocytes was regulated by p38 MAPK signaling. Taken together, our findings indicate that the activation of the C5a–C5aR1 axis in astrocytes facilitates the neuropathological changes resulting from EV-A71 infection, emphasizing the potential role of p38 MAPK-mediated CXCL1 production in these alterations.

**IMPORTANCE:**

Enterovirus A71 (EV-A71) is a common small RNA virus with highly neuroinvasive tendencies. Our previous studies took the view that EV-A71 could infect astrocytes and result in complement activation *in vivo*. We investigated how complement interacts with astrocytes to promote a severe inflammatory response upon EV-A71 infection in the study. As expected, our data demonstrate that EV-A71 triggers robust activation of the C5a–C5aR1 axis in astrocytes and that knockout or blockade of C5aR1 in animals exposed to lethal doses of EV-A71 significantly enhances survival by diminishing the production of the chemokines CXCL1 and IL-6. In addition, neutralizing CXCL1 significantly alleviates the neuropathogenesis caused by EV-A71 infection. Thus, inhibiting the C5a–C5aR1 axis has emerged as a potential therapeutic strategy to mitigate neural damage caused by EV-A71 infection.

## INTRODUCTION

Enterovirus A71 (EV-A71), a member of the genus *Enterovirus* of the *Picornaviridae* family, is an emerging neurotropic virus (1–3). Over the past 30 years, EV-A71 has been associated with escalating outbreaks of severe neurological disorders (e.g., encephalitis, meningitis, and acute flaccid paralysis) particularly affecting young children and infants globally, which has resulted in a substantial increase in mortality rates within this population (2, 4). In 1969, EV-A71 infections emerged in California, United States, demonstrating an association with sporadic instances of hand, foot, and mouth disease (HFMD), viral encephalitis, or a combination thereof (5). In 1997, a notable outbreak of EV-A71 infections characterized by viral encephalitis or acute flaccid paralysis was documented in Sarawak, Malaysia, with more than 2,600 cases and 41 fatalities (6). In 1998, Taiwan experienced a large-scale outbreak of EV-A71 infection, resulting in a total of 405 cases and 78 fatalities (7). From 2008 to 2015, several large-scale HFMD outbreaks occurred in Mainland China, with 13.8 million cases reported, including 130,000 severe cases with neurological diseases and 3,300 deaths. Among the reported patients with laboratory-confirmed diagnoses, 74% of patients with severe disease and 93% of patients who ultimately died tested positive for EV-A71 (8, 9). Since then, EV-A71 infection has spread widely in countries across the Asia–Pacific region, affecting millions of children per year (4). In 2013, Sydney, Australia, witnessed an outbreak of EV-A71 infection, leading to the diagnosis of more than 60 children with EV-A71-related neurological diseases (e.g., encephalomyelitis, brainstem encephalitis, and encephalitis) (10). To date, there is still no specific treatment for EV-A71 infection. Although an inactivated EV-A71 vaccine is available in China’s mainland (11) and Taiwan (12), the majority of the world, including North America, Australia, and Europe, must still address the ongoing threat posed by EV-A71.

Astrocytes, which are prevalent across the central nervous system (CNS), are the most abundant cell type and serve as pivotal contributors to diverse physiological functions and pathological processes within the CNS (13). Our previous studies (14, 15) suggested that EV-A71 infects mainly astrocytes in the brain and rarely infects neurons or microglia. However, the mechanism by which EV-A71 causes inflammatory damage in the brain is largely unknown. The complement cascade is a pivotal component of the innate immune system and is essential for effective defense against viral infections. However, excessive systemic activation of the complement system can result in adverse outcomes (16). The terminal products of complement activation include anaphylatoxins (C5a) and the terminal complement complex C5b-9. Notably, the interaction between C5a and its receptor, C5aR1, plays a pivotal role in driving inflammatory processes (17). In recent years, the physiological and pathological significance of complement biosynthesis in the CNS has received increasing attention (18). Complement factors are predominantly synthesized by the liver, and the entry of plasma proteins into the CNS is impeded by the blood–brain barrier (BBB). Nevertheless, most neural cells (e.g., astrocytes and microglia) have the ability to synthesize a comprehensive and functional complement system that encompasses complement factors, complement regulatory molecules, and complement receptors (17). There is growing evidence that the C5a–C5aR1 interaction is an important driver of neuroinflammation (16). Complement activation and C5a were detected in both viral encephalitis (tick-borne encephalitis) (19) and traumatic brain injury models (20). Currently, there are related drugs undergoing clinical trials for some complement-driven CNS diseases (21). Importantly, our proteomics research (22) revealed that EV-A71 induced alterations in the production of key proteins associated with the complement system in the brain. However, the role of the functional complement components within the complement system in the context of EV-A71 infection remains poorly understood.

Here, we used a well-established EV-A71 infection mouse model (23, 24) coupled with *in vitro* and human experiments to investigate complement activation in astrocytes after EV-A71 infection and to determine whether inhibition of the C5a–C5aR1 axis enhances survival. Our data demonstrate that EV-A71 triggers robust activation of the C5a–C5aR1 axis in astrocytes and that knockout or blockade of C5aR1 in animals exposed to lethal doses of EV-A71 significantly enhances survival by diminishing the production of the chemokines CXCL1 and IL-6. In addition, neutralizing CXCL1 significantly alleviates the neuropathogenesis caused by EV-A71 infection.

## MATERIALS AND METHODS

### Cell, treatment, and transfection

The U87-MG cell line, a human glioblastoma cell line, was obtained from the American Type Culture Collection. U87-MG cells were cultured in Dulbecco’s modified Eagle’s medium (DMEM) supplemented with 12% fetal bovine serum (Gibco) at 37°C. Primary astrocytes were prepared from C57BL/6J mice using a modification of technique (25). Within the initial 24 h after birth, C57BL/6J mice were sacrificed. The skin was incised, the skull was exposed, and the skull was carefully cut open to allow the extraction of intact brain tissue. After thorough washing with physiological saline, the brain was placed in precooled DMEM/F12. The meninges were removed from the brain, and the brain was carefully minced and digested with trypsin or papain. Following digestion, the tissue was filtered via a cell sieve, and the harvested cells were then cultured in flasks precoated with poly-L-lysine in a standard cell culture incubator. Astrocytes were separated at different time points by shaking at 200 rpm. Finally, cell purity was analyzed by flow cytometry.

For cell treatment, U87-MG cells were seeded in six-well plates at a density of 2 × 10^6^ cells per well, and then, 100 nM PMX53 or 100 nM PD169316 and EV-A71 were administered to the cells for 24 h treatment, following the manufacturer’s instructions.

For cell transfection, U87-MG cells were grown in six-well plates at a density of 2 × 10^6^ cells per well, and then, 100 nM siRNA was transiently transfected into the cells via Lipofectamine 2000.

### Virus and infection

The EV-A71 strain with the GenBank accession number OP806304 was isolated from a stool sample from a patient with nonfatal EV-A71 infection involving the CNS at Henan Children’s Hospital. The virus stock was propagated in RD cells, and virus titration and inoculation with EV-A71 were conducted as previously described (14, 23, 24). *In vitro*, U87-MG cells or primary nerve cells were infected with the virus at different multiplicities of infection (MOI). After 2 h, the unbound virus was removed, and the samples were further incubated at 37°C for 3, 6, 9, 12, and 24 h. The infected U87-MG cells were observed under a light microscope, and the cell viability was assessed via the MTT assay.

### Animal studies

C57BL/6J mice were acquired from Beijing Vital River Laboratory Animal Technology Co. Ltd., and C5aR1 knockout (KO) mice were initially obtained from the Jackson Laboratory. All the mice were kept under specific pathogen-free conditions in individually ventilated cages at the College of Public Health of Zhengzhou University. Following established protocols (23, 24), 5-d-old C57BL/6J mice or C5aR1 KO mice were intraperitoneally (i.p.) inoculated with 2.86 × 10^6^ TCID_50_ EV-A71 to establish the infection model. Mock-infected mice were i.p. inoculated with an equal volume of saline. For the isolation of mouse primary nerve cells, C57BL/6J mice at 7 days post infection (dpi) were anesthetized and the skull was removed to collect the brain. The brain was mechanically homogenated and filtered through 70 µm mesh to collect tissue precipitation. Brain homogenates were subjected to centrifugation using Percoll gradients, and the resulting cell populations were collected. The settings for the Percoll gradient are referenced from published research (26, 27). For the *in vivo* studies, at 5 d of age, C57BL/6J mice were intraperitoneally inoculated with PMX53/PMX53c or PD169316 at 2, 24, and 48 hours post infection (hpi) following lethal EV-A71 challenge. For *in vivo* neutralizing antibody treatment, 5-day-old C57BL/6J mice were i.p. inoculated with mouse anti-C-X-C motif chemokine ligand 1 (CXCL1), mouse anti-IL-6, or an isotype control 2 h before lethal EV-A71 challenge and 24 h and 48 h after viral challenge. The body weights, clinical manifestations, and survival of the mice were subsequently recorded daily until 15 dpi. Clinical scores were evaluated as described in our previous studies. The mice were euthanized under isoflurane anesthesia at 3, 5, and 7 dpi. The hearts of the mice were subsequently perfused with saline or paraformaldehyde to clear the blood, and the brains were preserved for subsequent studies (14, 22–24). The reagents used in the animal studies included PMX53/PMX53c (3.0 mg/kg bw), PD169316 (3.0 mg/kg bw), mouse αCXCL1 (3.0 µg/mouse), mouse αIL-6 (5.0 µg/mouse), and mouse IgG (3.0–5.0 μg/mouse).

### Study participants

This study included pediatric patients with HFMD who were hospitalized from April 2013 to September 2018. The diagnosis and classification of the patients adhered to the Guidelines for the Diagnosis and Treatment of Hand, Foot and Mouth Disease issued by the Ministry of Health in China. Patients who tested positive for EV-A71 were exclusively enrolled, and the parents or guardians of the participating children provided signed informed consent forms. EV-A71-infected patients who were diagnosed with encephalitis, aseptic meningitis, or encephalomyelitis were classified as having severe disease. In contrast, EV-A71-positive patients who did not experience organ damage and only experienced a rash were classified as having mild disease. Plasma samples from the participants were collected with EDTA and sodium citrate as anticoagulants. Following centrifugation at 3,000 rpm for 10 min (4°C), the supernatant was carefully aspirated, divided into 500 µL aliquots, and subsequently stored at −80°C for future use. Healthy control samples were obtained from the Biobank of Henan Children’s Hospital.

A paired case–control study was employed to identify specific biomarkers associated with EV-A71 infection. Samples from 18 healthy controls, 75 patients with mild EV-A71 infection, and 75 patients with severe EV-A71 infection were included in the complement factor and cytokine analysis. Finally, we created a model via multivariate logistic regression to assess the predictive value of complement components for severe illness. The demographic information and clinical laboratory results of the participants are presented in Table S1.

### ELISA

Human plasma C1q, C3a, C3b, FB, C5a, GFAP, and CXCL1 levels were determined via enzyme-linked immunosorbent assay (ELISA) kits purchased from Cusabio Biotechnology Inc. U87-MG cells were plated in 96-well plates and infected with EV-A71 for different time intervals. Next, the cell supernatants were harvested. The levels of IL-6, CXCL1, and TNF-α were assessed via ELISA kits purchased from BioLegend Inc. C3 and C5a concentrations in mouse brain tissue homogenates were determined via ELISA kits purchased from Cusabio Biotechnology Inc. All procedures were meticulously executed in strict accordance with the manufacturer’s instructions.

### Confocal microscopy

U87-MG cells (1 × 10^4^) were seeded on 35-mm laser confocal petri dishes (with a bottom diameter of 10 mm). After treatment with EV-A71 for different durations, the cells were washed with phosphate buffer saline (PBS), fixed in 4% formaldehyde for 30 min, and subsequently blocked and permeabilized with Triton X-100 for 10 min at room temperature. The cells were incubated for 1 h with primary antibodies at room temperature, followed by incubation with Cy3-conjugated goat anti-rabbit/mouse IgG. The cell nuclei were stained with DAPI for 5 min. The stained cells were examined via a Leica TSC SP8 confocal laser scanning microscope.

### Western blot analysis

For the preparation of whole-cell lysates from both U87-MG cells and animal tissues, RIPA lysis buffer supplemented with proteinase and phosphatase inhibitors was utilized. Lysis was carefully conducted on ice for 30 min to 1 h. Then, the supernatant was collected by centrifugation at 12,000 × *g* for 10 min. To accurately determine the protein concentration for subsequent analyses, a BCA assay kit was used. The proteins were separated via SDS–PAGE and subsequently transferred to a PVDF membrane. After being blocked with skim milk, the membrane was incubated with antibodies, followed by development via an enhanced chemiluminescence kit. The protein bands were imaged via an Amersham Imager 600 imaging system and analyzed via ImageJ software.

### Histopathology

Brain tissues were carefully collected, fixed in paraformaldehyde, and subsequently embedded in paraffin. The resulting paraffin-embedded tissues were sectioned into 5-µm slices, followed by staining with hematoxylin-eosin (H&E) for subsequent histopathological examination. The sections were scored in a blinded manner, adhering to an established protocol (28). Immunofluorescence (IF) staining was employed for the detection of neutrophils following previously described procedures (23) by Servicebio Biotech Co. Ltd. Representative images were captured via SlideViewer advanced slide viewing software from 3DHISTECH.

### RNA extraction, reverse transcription, and qRT-PCR analysis

Total RNA was extracted via TRIzol reagent and then was converted to single-stranded DNA with the cDNA Synthesis SuperMix Kit. Quantitative real-time PCR (qRT-PCR) was conducted via the use of SYBR Green Master Mix on the CFX Opus 96 Real-Time PCR System. All reactions were performed in triplicate, and the analysis was executed via the 2^−ΔΔCt^ method. The sequences of primers used for qRT-PCR are listed in Tables S2 and S3 .

### Determination of viral RNA copy number

The quantification of EV-A71 genomic RNA was carried out through qRT-PCR using a primer pair. The resulting 226 bp fragment, amplified by the VP1 primers, was incorporated into the pET-28a (+) plasmid, generating the pET-28a (+)-EV-A71 VP1 plasmid. This plasmid served as a standard for quantifying the EV-A71 copy number. A standard curve was generated through serial dilutions of pET-28a (+)-EV-A71 VP1, and linear regression was applied, resulting in the following equation: *Y* = −0.3164*X* + 12.939, where *X* represents the CT value and *Y* represents log_10_. A high coefficient of determination (*R*^2^ = 0.9984) indicated the reliability of the standard curve. Viral loads were computed and are presented as log_10_ (viral RNA copies) per milligram of tissue.

### Analysis of brain cells by FACS

Brain was transferred to a tissue grinder filled with 1640 buffer and thoroughly ground. Then, the tissue was passed through a 75 µm filter to separate the cells and tissue fragments in the cell suspension. The brain homogenate was collected, centrifuged at 450 × *g* for 5 min, resuspended in 2 mL ACK lysis buffer, and incubated on ice to ensure complete lysis of the red blood cells. Next, the cells were incubated with an anti-mouse CD16/32 antibody at 4°C for 20 min, and dead cells were excluded via live/dead staining. After being washed with 1× hank's balanced salt solution (HBSS) buffer, the cells were resuspended in 50 µL of buffer containing fluorescently labeled monoclonal antibodies and incubated for 25 min. Intracellular antibodies were incubated with IC Fixation Buffer for 30 min, followed by incubation with 1× permeabilization buffer. Finally, the cells were fixed with 2% paraformaldehyde. Flow cytometry was performed via Agilent NovoCyte, and subsequent data analysis was performed with FlowJo Software. Astrocyte abundance was determined by staining for the astrocyte activation marker GFAP.

### Antibodies

The following antibodies were applied for immunofluorescence (IF) staining and western blot analysis: anti-EV-A71 3D, EV-A71 3C, and EV-A71 VP1 (GeneTex Inc., California, USA); anti-p38, pp38 (Thr180/Thr182), NF-κB p65, pp65 (Ser536), Caspase-3, and Cleaved-Caspase-3 (Cell Signaling Technologies Inc., Danvers, Massachusetts, USA); anti-IBA1, NeuN, GFAP, Ly6G, HRP-Goat anti-Rabbit lgG, Cy3-conjugated Goat anti-Rabbit IgG, Cy3-conjugated Goat anti-Mouse IgG, FITC-conjugated Goat anti-Rabbit IgG, and FITC-conjugated Goat anti-Mouse IgG (Servicebio Biotech Co. Ltd., Wuhan, China); anti-GFAP, C1q, C3, C5aR, C5a/C5a des Arg, GAPDH, and β-actin (Abcam Biotechnology Inc., Cambridge, UK); anti-phosphor-C5aR1 (Ser327) and APC anti-Mouse/Human p-p38 (Thr180/Tyr182) (Invitrogen Inc., USA); and APC anti-Mouse CD88 (C5aR), PerCP/Cyanine5.5 anti-Mouse CD45, Pacific Blue anti-Mouse/Human CD11b, and PE Donkey anti-Rabbit IgG (minimal x-reactivity) (BioLegend Inc., Japan).

### Statistical analysis

The means with interquartile ranges, means with standard deviations, and proportions (%) were computed and analyzed via either SPSS (version 29.0, IBM, USA) or GraphPad Prism software. The Mann–Whitney U test or Student’s *t* test was used to compare two datasets. The χ^2^ test was employed for categorical variables. Pearson’s correlation was used to explore the associations between two variables. Survival analysis was conducted via the Kaplan–Meier method, and any disparities in survival were evaluated with a stratified log-rank test. For all analyses, differences were considered significant at *P* < 0.05.

## RESULTS

### EV-A71 predominantly infects astrocytes and causes astrocyte activation

**Fig 1:**
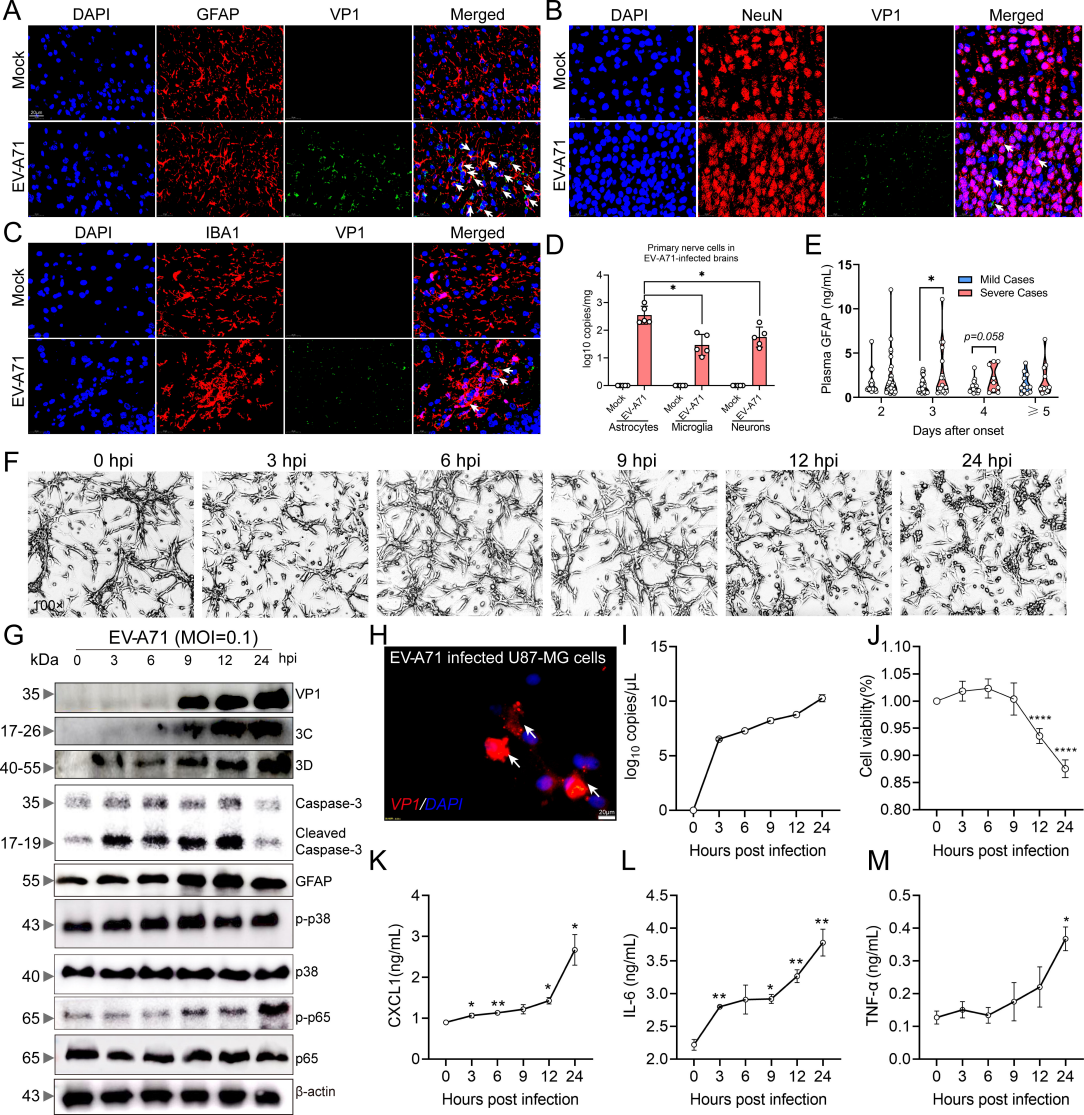
EV-A71 tends to infect astrocytes and leads to astrocyte activation. Five-day-old C57BL/6J mice were intraperitoneally inoculated with 2.86 × 10^6^ TCID_50_ EV-A71 or saline, and sacrificed at 7 dpi. We measured the amount of co-localization between astrocytes (GFAP) and EV-A71 (**A**), neurons (NeuN) and EV-A71 (**B**), microglia (IBA1), and EV-A71 (**C**) in brain by immunofluorescence staining. The presentative images were obtained using SlideViewer, 3DHISTECH’s advanced slide viewing software. Bar = 20 µm. Primary astrocytes, microglia, and neurons were isolated from EV-A71-infected mice at 7 dpi, and the viral loads in various types of nerve cells were detected by qRT-PCR (*n* = 5) (**D**). The concentration of GFAP (**E**) in mild (*n* = 75) and severe cases (*n* = 75) with EV-A71 infection was determined by ELISA. U87-MG cells were cultured at a density of 2 × 10^6^/well in specialized petri dishes, and we photographed the state of the cells post-EV-A71 infection (MOI = 0.1) under a microscope. The magnification is to be 100× (**F**). The expression of VP1, 3C, 3D, Cleaved Caspase-3, and GFAP and phosphorylation levels of p38 MAPK and NF-κB p65 in total lysates of U87-MG cells were analyzed (**G**). Intracellular localization of VP1 (**H**) was evaluated by immunofluorescence staining (DAPI: blue; VP1: red). 1 × 10^4^ U87-MG cells were seeded in 96-well plate, and then, virus was added for infection (MOI = 0.1). After different time intervals of infection, viral loads (**I**) were quantified by qRT-PCR, and cell viability was determined by MTT assay (**J**). The concentrations of CXCL1 (**K**), IL-6 (**L**), and TNF-α (**M**) in the cell supernatants were determined using the ELISA technique. White arrows indicate EV-A71-infected cells. The immune blots presented in this figure are derived from three replicates of representative images. DAPI, 4’,6-Diamidino-2-phenylindole. *P ＜0.05 vs mild/0 hpi (*n* = 3), **P ＜0.01 vs 0 hpi (*n* = 3), and ****P ＜0.0001 vs 0 hpi (*n* = 3).

Initially, we used immunofluorescence staining to observe the distribution of EV-A71 in the brain at 7 dpi (Fig. 1A, B and C) and further identified astrocytes as the primary target cells for EV-A71 infection by measuring viral loads in various types of nerve cells (Fig. 1D). To underscore the clinical significance of our findings, we determined the plasma GFAP concentration in both patients with mild EV-A71 infection and patients with severe EV-A71 infection (who had encephalitis) EV-A71. We observed that the plasma GFAP concentration in patients with severe infection was significantly greater than that in patients with mild infection, and this change seemed to occur on day 3 after onset (Fig. 1E). Next, to confirm whether EV-A71 replicates in astrocytes, we used U87-MG cells as an *in vitro* model. As shown in Fig. 1F, EV-A71 led to cytopathic effects on U87-MG cells, including a reduction in synapse number, rounding, and shedding. Furthermore, the western blot results revealed that EV-A71 replication in astrocytes was characterized by an increase in VP1, 3C, 3D, and Cleaved Caspase-3 levels, and our data also indicated that EV-A71 led to astrocyte activation characterized by an increase in GFAP levels and the phosphorylation of p38 and NF-κB p65 (Fig. 1G and H). The viral load gradually increased (Fig. 1I), whereas the cell viability gradually decreased (Fig. 1J), in EV-A71-infected U87-MG cells as the duration of infection increased. EV-A71 infection triggered substantial upregulation of various inflammatory cytokines. Nevertheless, the specific impact of inflammatory cytokines on infection outcomes within the CNS remained unclear. Thus, we measured the concentrations of CXCL1, IL-6, and TNF-α in the culture supernatants of EV-A71-infected U87-MG cells and found that the levels of these three cytokines were significantly increased at 24 hpi (Fig. 1K, L and M). Taken together, our findings suggest that EV-A71 predominantly infects astrocytes and causes astrocyte activation.

### The C5a–C5aR1 axis mediates astrocyte activation upon EV-A71 infection

**Fig 2:**
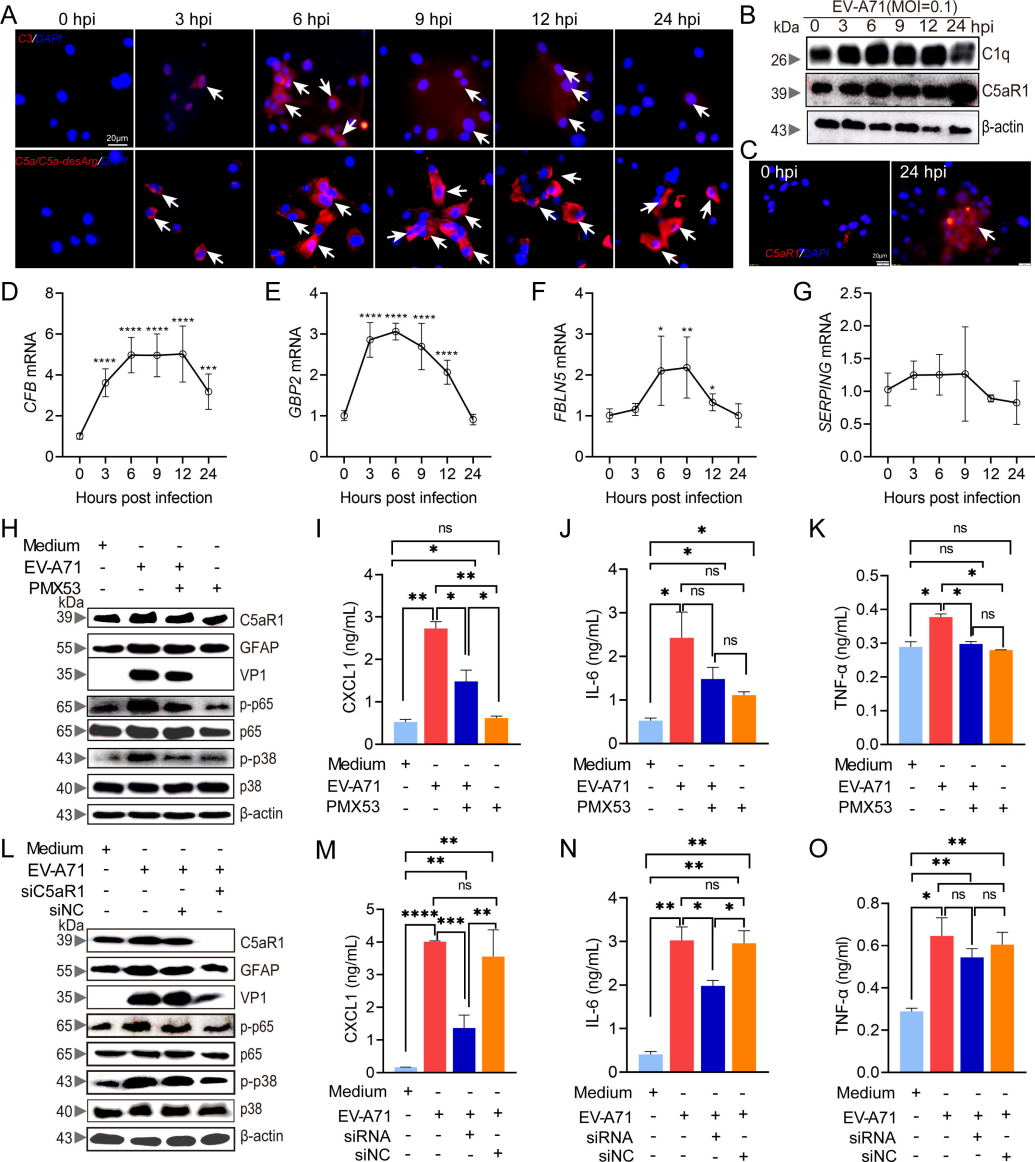
The regulatory role of the C5a-C5aR1 axis in astrocyte activation. U87-MG cells (2 × 10^6^) were seeded on laser confocal petri dishes or in a six-well plate, and virus was added for infection. After different time intervals of infection, the expression of C3 (**A**), C5a/C5a-desArg (**A**), and C5aR1 (**C**) was conducted with confocal laser scanning microscopes, and the protein expression for C1q and C5aR1 (**B**) was evaluated by western blot, and the mRNA expression levels for *CFB* (**D**), *GBP2* (**E**), *FBLN5* (**F**), and *SERPING* (**G**) were analyzed by qRT-PCR. U87-MG cells were seeded in petri dish, and the virus (MOI = 0.1), PMX53 (100 nM), and siC5aR1 (100 nM) were added. After 24 h of treatment, western blot analysis was conducted on cell lysates using different antibodies (**H, L**). The indicated values represent the relative intensity of the proteins normalized to β-actin. As mentioned above, U87-MG cells were meticulously plated in a 96-well plate at a density of 1 × 10^4^ cells per well, followed by treatment with EV-A71 (MOI = 0.1), PMX53 (100 nM), siC5aR1 (100 nM), and negative control for different time intervals. After 24 h, the cell supernatants were harvested for cytokine release assays (**I–K, M–O**). White arrows indicate positive-stained cells. The immune blots presented in this figure are derived from three replicates of representative images. *P ＜0.05 vs 0 hpi (*n* = 3), **P ＜0.01 vs 0 hpi (*n* = 3), ***P ＜0.001 vs 0 hpi (*n* = 3), and ****P ＜0.0001 vs 0 hpi (*n* = 3).

Our previous study suggested that complement activation occurs in the mouse brain upon EV-A71 infection (29). To investigate whether EV-A71 infection leads to complement activation in astrocytes, the levels of complement components in U87-MG cells were measured. As depicted in Fig. 2A through C, the expression levels of C1q, C3, C5a/C5a-desArg, and C5aR1 were elevated at different time points after EV-A71 infection. Similarly, the expression of complement-related genes (*CFB*, *GBP2*, and *FBLN5*) was significantly changed (Fig. 2D through G). The activation of the C5a–C5aR1 axis serves as a crucial catalyst for neuroinflammation. To further elucidate the role of the C5a–C5aR1 pathway in astrocyte activation and inflammatory cytokine production in response to EV-A71 infection, we used PMX53 and siRNA to repress the expression of C5aR1. As shown in Fig. 2H, PMX53 treatment reduced the expression of C5aR1 in U87-MG cells and inhibited astrocyte activation, p38 and NF-κB p65 phosphorylation, and VP1 expression. Furthermore, the levels of CXCL1 (Fig. 2I), IL-6 (Fig. 2J), and TNF-α (Fig. 2K) in the culture supernatants were increased by EV-A71 infection. However, after PMX53 treatment, the levels of TNF-α and CXCL1 were significantly reduced, suggesting that C5aR1 is involved in the production of these cytokines. As shown in Fig. 2L, similar results were obtained by siC5aR1 transfection in U87-MG cells, and a corresponding decrease in the production of cytokines, especially CXCL1 and IL-6, was observed (Fig. 2M through O). Together, our results demonstrate that the C5a–C5aR1 axis is required for EV-A71-mediated induction of proinflammatory cytokines in astrocytes.

### Activation of the C5a–C5aR1 axis in astrocytes is essential for neuropathogenesis caused by EV-A71 infection

**Fig 3:**
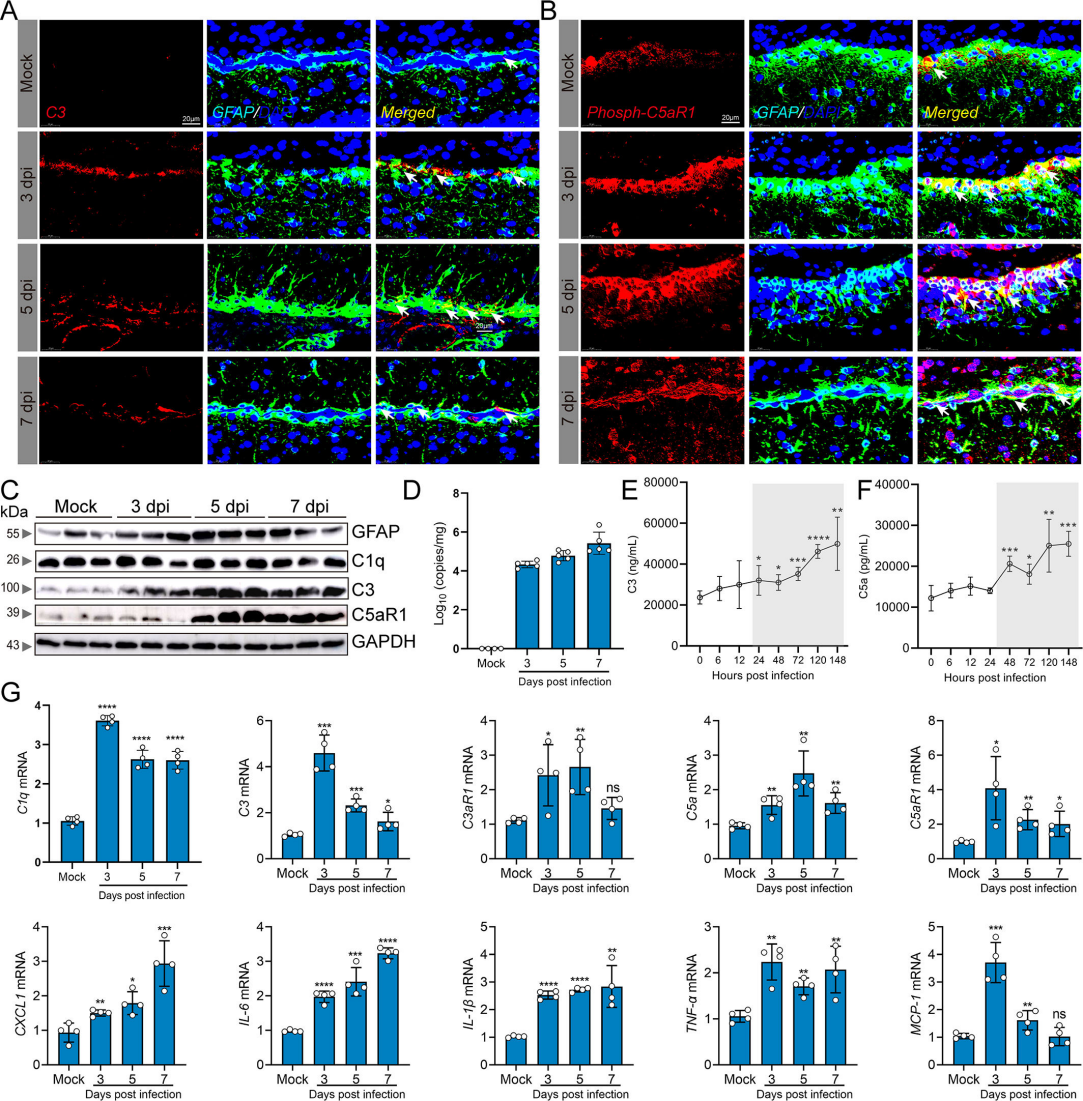
Complement activation during EV-A71-induced neuropathogenesis. Five-day-old C57BL/6J mice were intraperitoneally inoculated with either 2.86 × 10^6^ TCID_50_ EV-A71 or saline (mock) and sacrificed at 3, 5, and 7 dpi. Complement activation in brain astrocytes was assessed by immunofluorescence co-localization (**A, B**). Red fluorescence represents C3 or phosphor-C5aR1 (Ser327), blue fluorescence represents the nucleus, and green fluorescence represents astrocytes. The expression levels of GFAP, C1q, C3, and C5aR1 in a whole-brain lysate (**C**) were quantified by western blot. Viral loads (**D**) in the brain were quantified by qRT-PCR. Temporal fluctuations in the protein levels of C3 (**E**) and C5a (**F**) in the brain were assessed using ELISA kit. The mRNA levels of complement-related genes and inflammatory cytokines (**G**) were determined by qRT-PCR. White arrows indicate double-positive cells. *P ＜0.05 vs mock/0 hpi (*n* = 3–5), **P ＜0.01 vs mock/0 hpi (*n* = 3–5), ***P ＜0.001 vs mock/0 hpi (*n* = 3–5), and ****P ＜0.0001 vs mock/0 hpi (*n* = 3–5).

Given that astrocyte activation is commonly linked with neuroinflammation, we subsequently explored the pivotal role of the C5a–C5aR1 axis in the development of encephalitis resulting from EV-A71 infection. To this end, we assessed complement activation in astrocytes via different assay techniques. Through immunofluorescence staining, we found that C3 (Fig. 3A) and phospho-C5aR1 (Ser327) (Fig. 3B) were strongly colocalized in astrocytes and that there was a significant increase in C3 and phospho-C5aR1 levels in EV-A71-infected mice compared with uninfected mice at 3, 5, and 7 dpi. Through western blot analysis, we observed that EV-A71 infection resulted in the upregulation of GFAP, C3, and C5aR1 expression in a time-dependent manner (Fig. 3C). Figure 3D showed the changes in viral load at 3, 5, and 7 dpi. Following EV-A71 infection, a rapid and dramatic increase in brain C3 and C5a levels was observed, peaking at 148 hpi (Fig. 3E and F). Fluorescence-activated cell sorting (FACS) analyses confirmed activation of the C5a–C5aR1 axis in astrocytes (Fig. S1). Moreover, we observed that EV-A71 infection increased C1q, C3, C3aR1, C5a, and C5aR1 mRNA levels and the transcription levels of proinflammatory cytokines, including CXCL1, IL-6, IL-1β, TNF-α, and MCP-1, within brain tissue (Fig. 3G). Collectively, these findings suggest that the activation of the C5a–C5aR1 axis in astrocytes may contribute to the neuropathological changes caused by EV-A71 infection.

**Fig 4:**
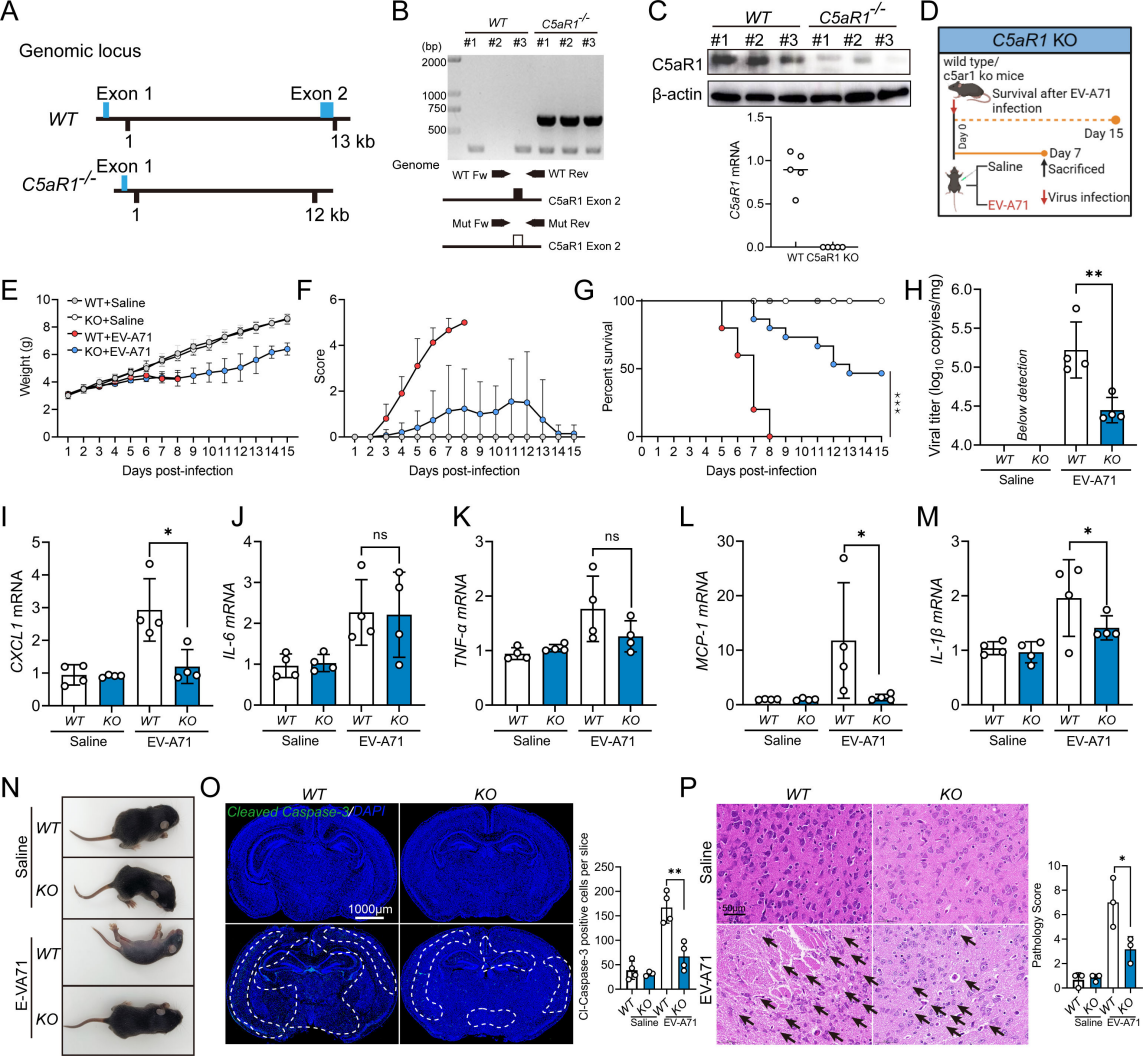
C5aR1 promotes EV-A71-induced neuropathogenesis. (**A**) The genomic locus map of the C5aR1 gene in WT and C5aR1 KO (C5aR1^−/−^) mice. Genomic DNAs were extracted from individual mouse brain tissues, and the deletion of the C5aR1 gene in the genome was identified through PCR and agarose gel electrophoresis using specific primers as detailed below (**B**). Mutant (C5aR1 deletion) (Mut Fw): 5′-CCTGCCAGAGGACAGGAGT-3′; mutant (Mut Rev): 5′-CATGCTGGGATATAGGTTTGCGC-3′. The lengths of products are 700–1000 bp for mutant. The confirmation of C5aR1 protein and mRNA expression in brain tissue was meticulously carried out via western blot and qRT-PCR (**C**). (**D**) Five-day-old WT/KO mice were i.p. inoculated with 2.86 × 10^6^ TCID_50_ EV-A71 or saline. A daily record was kept on the body weight (**E**), clinical manifestations (**F**), and survival rates (**G**) of mice until 15 dpi (*n* = 6–15). Tissue analysis was performed on day 7 post-infection. (**H**) Viral load in brain tissue (*n* = 5). The mRNA levels of CXCL1 (**I**), IL-6 (**J**), TNF-α (**K**), MCP-1 (**L**), and IL-1β (**M**) in mouse brain were quantified by qRT-PCR at 7 dpi (*n* = 4). (**N**) The presentative images of mice on 7 dpi in the specified groups. Cleaved Caspase-3 immunofluorescence staining (**O**) and H&E staining (**P**) were conducted to assess brain pathology (*n* = 3). The white-dotted areas indicate where apoptosis mainly occurs, and the black arrows indicate neuronal damage and degeneration. *P ＜0.05 vs WT mice, **P ＜0.01 vs WT mice, and ****P ＜0.0001 vs WT mice.

To further explore the involvement of the C5a–C5aR1 axis in EV-A71 encephalitis, we generated C5aR1-KO mice via the *CRISPR/Cas9* system. According to the C5aR1 sequence, C5aR1 has multiple transcription products. Deletion of exon 2 of C5aR1 results in loss of function of the entire gene, generating a mutant phenotype. Therefore, we designed guide RNAs targeting both sides of exon 2 to delete an approximately 1,053 bp region and thus knockout C5aR1 (Fig. 4A and B). As expected, C5aR1 was detected in the primary astrocytes of wild type (WT) mice but was absent in the primary astrocytes of C5aR1^−/−^ mice (Fig. 4C). Knockout of C5aR1 led to an apparent reduction in susceptibility to EV-A71 infection in mice, as illustrated by the alleviation of clinical symptoms (Fig. 4D through F and N) and an increase in survival rate (Fig. 4G), as well as significant reductions in viral load (Fig. 4H) and proinflammatory cytokine (CXCL1, IL-1β, and MCP-1) (Fig. 4I through M) levels in these mice upon viral infection. Additionally, a reduction in the number of Cleaved-Caspase-3-positive cells (Fig. 4O) and a decrease in the degree of neuronal damage (Fig. 4P) were observed in C5aR1^−/−^ mice, which was consistent with the above observations. Taken together, our findings highlight that the activation of the C5a–C5aR1 pathway in astrocytes facilitates EV-A71-induced neuropathogenesis.

### Inhibition of the C5a-C5aR1 axis mitigates mouse susceptibility to EV-A71 infection

**Fig 5:**
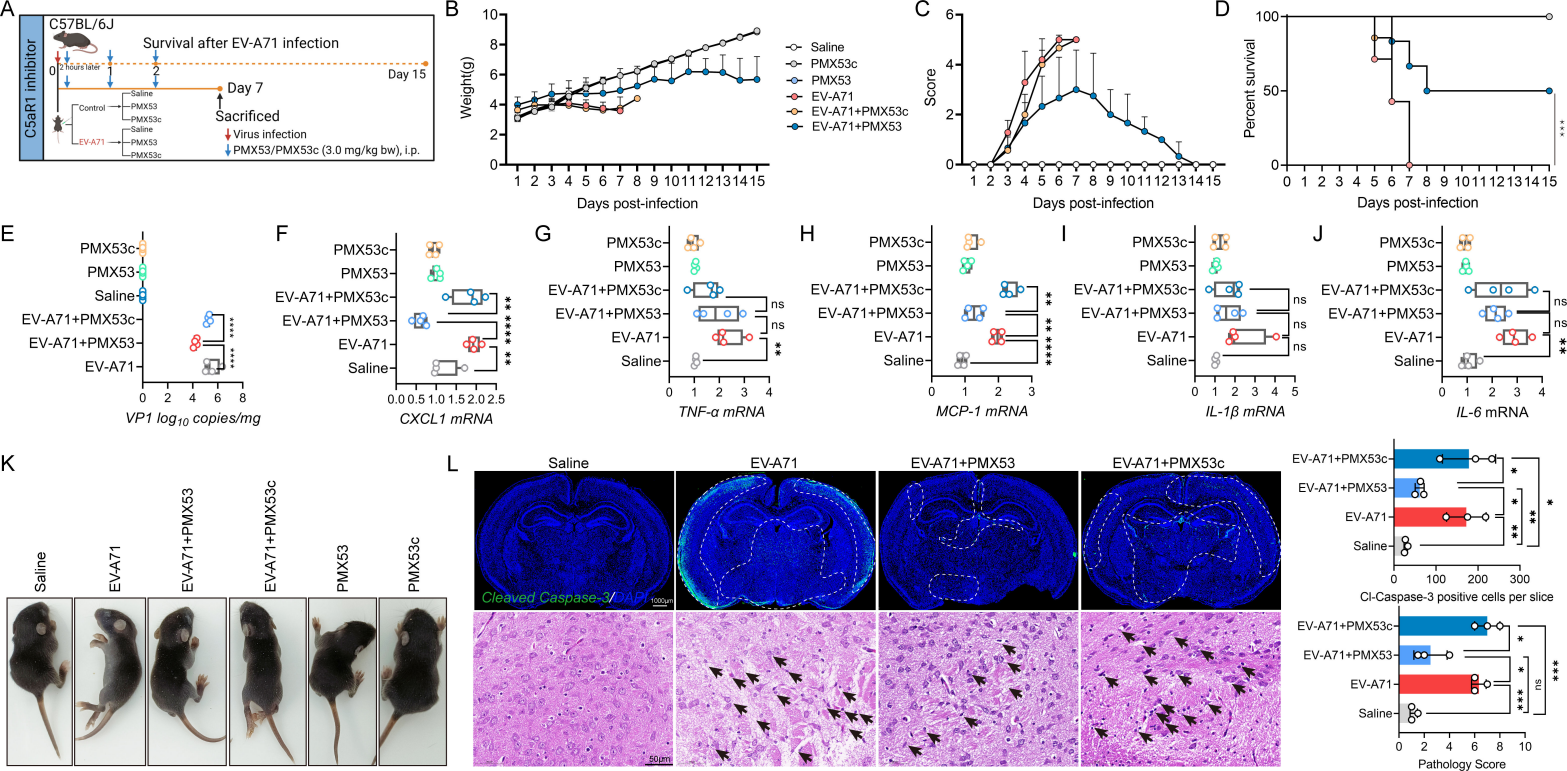
Complement inhibition with C5aR1 antagonist attenuated brain injury after EV-A71 challenge. (**A**) Five-day-old C57/BL6J mice upon EV-A71 infection were treated with PMX53 (3.0 mg/kg bw) in the schedule and sacrificed at 7 dpi. The body weight (**B**), clinical scores (**C**), and Kaplan–Meier survival curve (**D**) were meticulously recorded from 1 to 15 dpi (*n* = 6–11). (**E**) Viral load in brain tissue (*n* = 5–6). The mRNA levels of CXCL1 (**F**), TNF-α (**G**), MCP-1 (**H**), IL-1β (**I**), and IL-6 (**J**) in mouse brain were quantified by qRT-PCR at 7 dpi (*n* = 4). (**K**) The presentative images of mice at 7 dpi in the specified groups. Cleaved Caspase-3 immunofluorescence staining and H&E staining (**L**) were conducted to assess brain pathology (*n* = 3). The white-dotted areas indicate where apoptosis mainly occurs, and the black arrows indicate neuronal damage and degeneration. *P ＜0.05 vs indicated group, **P ＜0.01 vs indicated group, ***P ＜0.001 vs indicated group, and ****P ＜0.0001 vs indicated group.

To assess the potential therapeutic efficacy of targeting C5aR1 in EV-A71 encephalitis, the C5aR1 antagonist PMX53 was used for subsequent experiments. PMX53 (3D53) is a synthetic peptide that serves as a potent and orally active antagonist of the complement C5aR (CD88). PMX53 exhibits a specific binding affinity for C5aR1, while showing no interaction with the second C5a receptor (C5L2) or C3aR. Furthermore, PMX53 demonstrates notable anti-inflammatory properties. Figure 5A shows the experimental flowchart. We observed that administering PMX53 to WT mice infected with the virus provided a protective effect equivalent to that of C5aR1 knockout. As shown in Fig. 5B through D and K, PMX53 administration attenuated clinical signs and scores and significantly improved the survival of infected mice. Additionally, PMX53 administration significantly reduced the viral load in brain tissue (Fig. 5E). We subsequently investigated the modulatory effect of PMX53 on proinflammatory cytokine production following EV-A71 infection. Our results revealed that inhibition of the C5a–C5aR1 axis inhibited the EV-A71-induced inflammatory response (Fig. 5F through J). Consistent with the above results, the degree of brain damage also significantly decreased after the administration of PMX53 (Fig. 5L). Therefore, our findings demonstrate that targeting C5aR1 has potential therapeutic value for treating EV-A71 encephalitis.

### Complement activation in children with EV-A71 encephalitis

**Fig 6:**
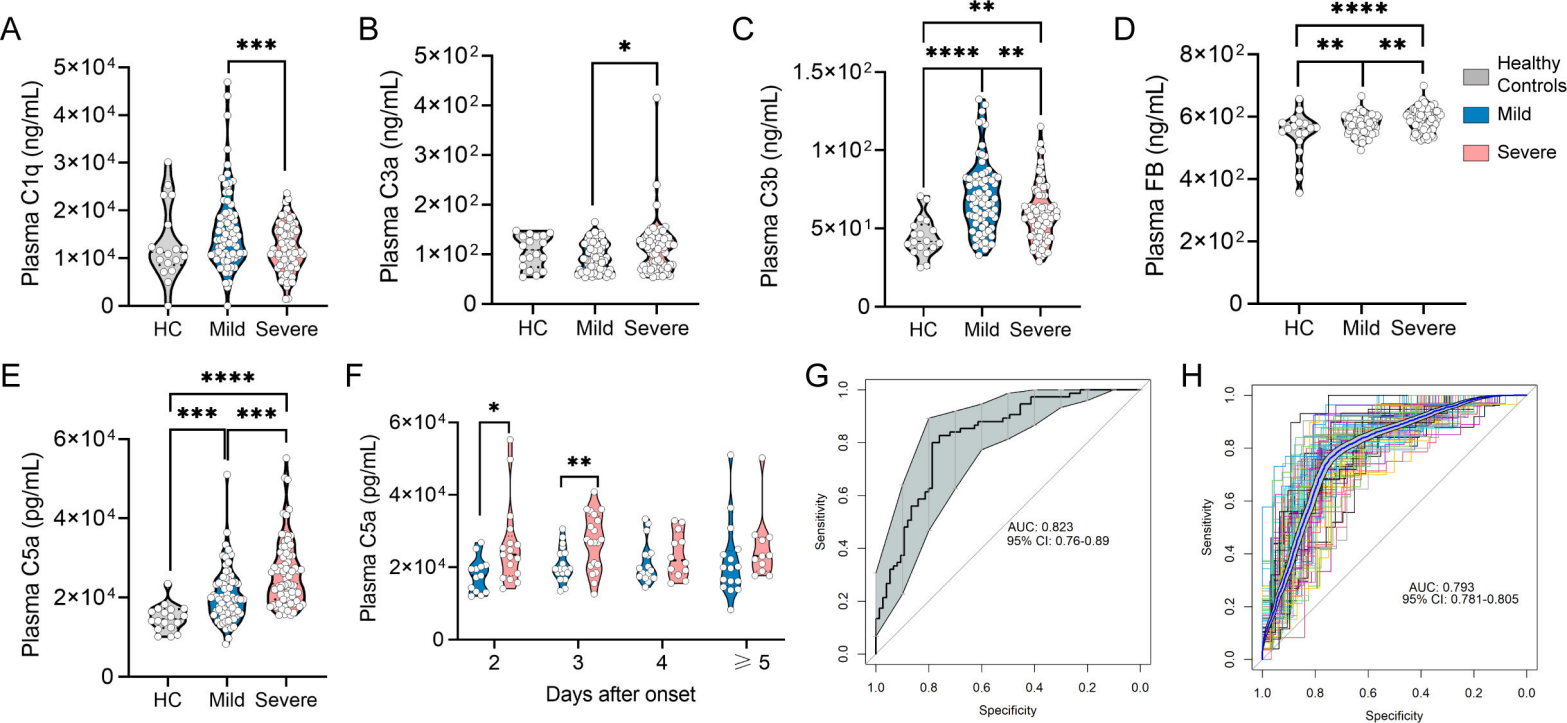
Expression levels of complements in the plasma of EV-A71-infected patients. The concentrations of C1q (**A**), C3a (**B**), C3b (**C**), FB (**D**), and C5a (**E**) were measured by ELISA kit. (**F**) Changes in plasma C5a in EV-A71-infected patients with different onset times. (G) Logistic regression was employed to establish a comprehensive predictive model for severe illness, incorporating quantitative parameters that demonstrated statistical significance. (**H**) Leave-one-out cross-validation. *P ＜0.05 vs indicated group, **P ＜0.01 vs indicated group, ***P ＜0.001 vs indicated group, and ****P ＜0.0001 vs indicated group.

To assess the clinical relevance of C5a–C5aR1 axis activation in EV-A71 infection, we analyzed plasma complement component levels in individuals with mild and severe EV-A71 infection, as well as in healthy controls. Our results revealed a notable reduction in the levels of C1q (Fig. 6A) and C3b (Fig. 6C) in the plasma of patients with severe infection compared with those with mild infection, suggesting depletion of complement components. Importantly, the levels of C3a (Fig. 6B), FB (Fig. 6D), and C5a (Fig. 6E) in the plasma of patients with severe infection were significantly greater than those in the plasma of patients with mild infection and healthy controls. We also observed an increase in the plasma levels of C5a from the second day to the third day after infection onset (Fig. 6F). We subsequently constructed a predictive model that integrated four parameters through logistic regression. Figure 6G shows the receiver operating characteristic (ROC curve), along with the area under the curve (AUC) and its 95% confidence interval (CI), for both the selected parameters and the model, and the leave-one-out analysis of the cross-validation data revealed that the AUC based on logistic regression was 0.793 (Fig. 6H). Overall, these findings indicate that the C5a–C5aR1 axis is activated in children with EV-A71 encephalitis and that complement activation has important prognostic value in patients with EV-A71 infection.

### CXCL1 is synthesized predominantly by astrocytes

**Fig 7:**
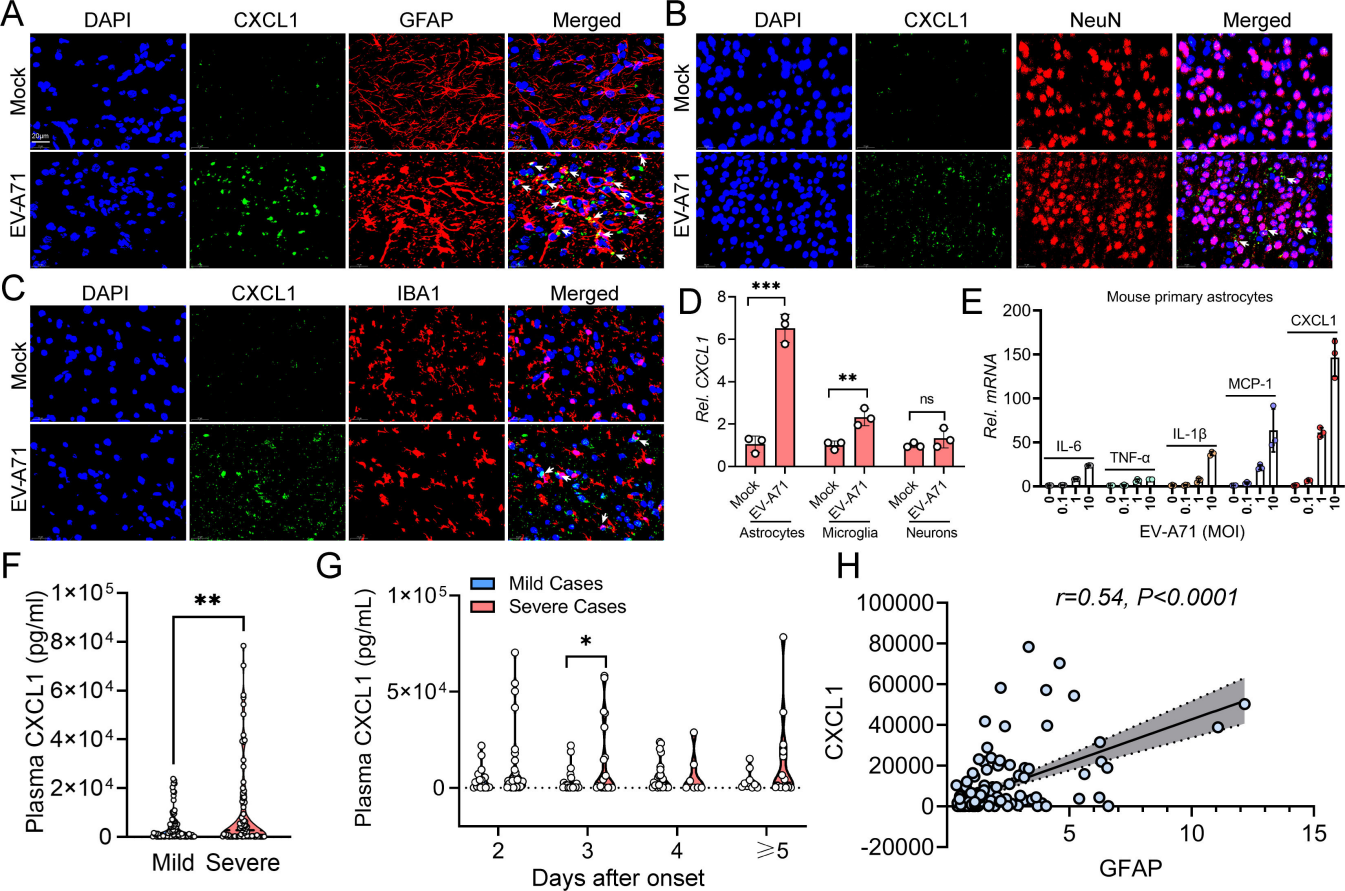
The main source of CXCL1 during EV-A71-induced neuropathogenesis. Five-day-old C57BL/6J mice were intraperitoneally inoculated with 2.86 × 10^6^ TCID_50_ EV-A71 or saline and sacrificed at 7 dpi. Co-localization of GFAP+ astrocytes (**A**), NeuN+ neurons (**B**), and IBA1+ microglia (**C**) with CXCL1 was assessed through immunofluorescence staining (*n* = 3). (**D**) The mRNA expression of CXCL1 in primary astrocytes, microglia, and neurons isolated from EV-A71-infected mice at 7 dpi was detected by qRT-PCR (*n* = 3). Mouse primary astrocytes (2 × 10^6^) were cultured in a six-well-plate and were exposed to various MOIs of EV-A71 for a duration of 24 h. (**E**) qRT-PCR was employed to assess the mRNA expression levels of IL-6, CXCL1, TNF-α, MCP-1, and IL-1β (*n* = 3). The concentration of CXCL1 (**F**) in the plasma of EV-A71-infected patients was measured by ELISA. (**G**) Changes in plasma CXCL1 in EV-A71-infected patients with different onset times. (**H**) Correlation analysis of plasma GFAP and CXCL1. White arrows indicate double-positive cells. *P ＜0.05 vs indicated group and **P ＜0.01 vs indicated group.

CXCL1 expression in the CNS has been demonstrated to be associated with breakdown of the BBB and the development of viral encephalitis (30). According to the above findings, CXCL1 expression was most affected by C5aR1 blockade or knockout. Following EV-A71 infection, we further investigated the glial populations associated with CXCL1 production by preparing whole-brain tissues for confocal microscopy. In addition to staining for CXCL1, brain slices were subjected to staining for GFAP to specifically label astrocytes, IBA1 for microglia identification, and NeuN for neuronal labeling. As shown in Fig. 7A through C, CXCL1 was mainly localized in perivascular GFAP+ astrocytes and a limited number of IBA1+ microglia and NeuN+ neurons. To validate these findings, we evaluated the transcription levels of CXCL1 and 4 cytokines in primary astrocytes, microglia, and neurons from EV-A71-infected mice at 7 dpi (Fig. 7D; Fig S2). The expression of chemokines and cytokines, especially CXCL1 in astrocytes (Fig. 7D and E), was markedly induced after EV-A71 infection. To further investigate whether CXCL1 production was associated with astrocyte activation in the study participants, we measured the levels of CXCL1 in the plasma of EV-A71-infected children (Fig. 7F and G). We found that plasma CXCL1 levels were significantly elevated in patients with severe EV-A71 infection on the third day after infection onset (Fig. 7G). Correlation analysis revealed a positive association between GFAP levels and CXCL1 expression (Fig. 7H). Together, these results suggest that CXCL1 is produced mainly by astrocytes.

### Neutralization of CXCL1 *in vivo* attenuates EV-A71-induced neuropathogenesis changes

**Fig 8:**
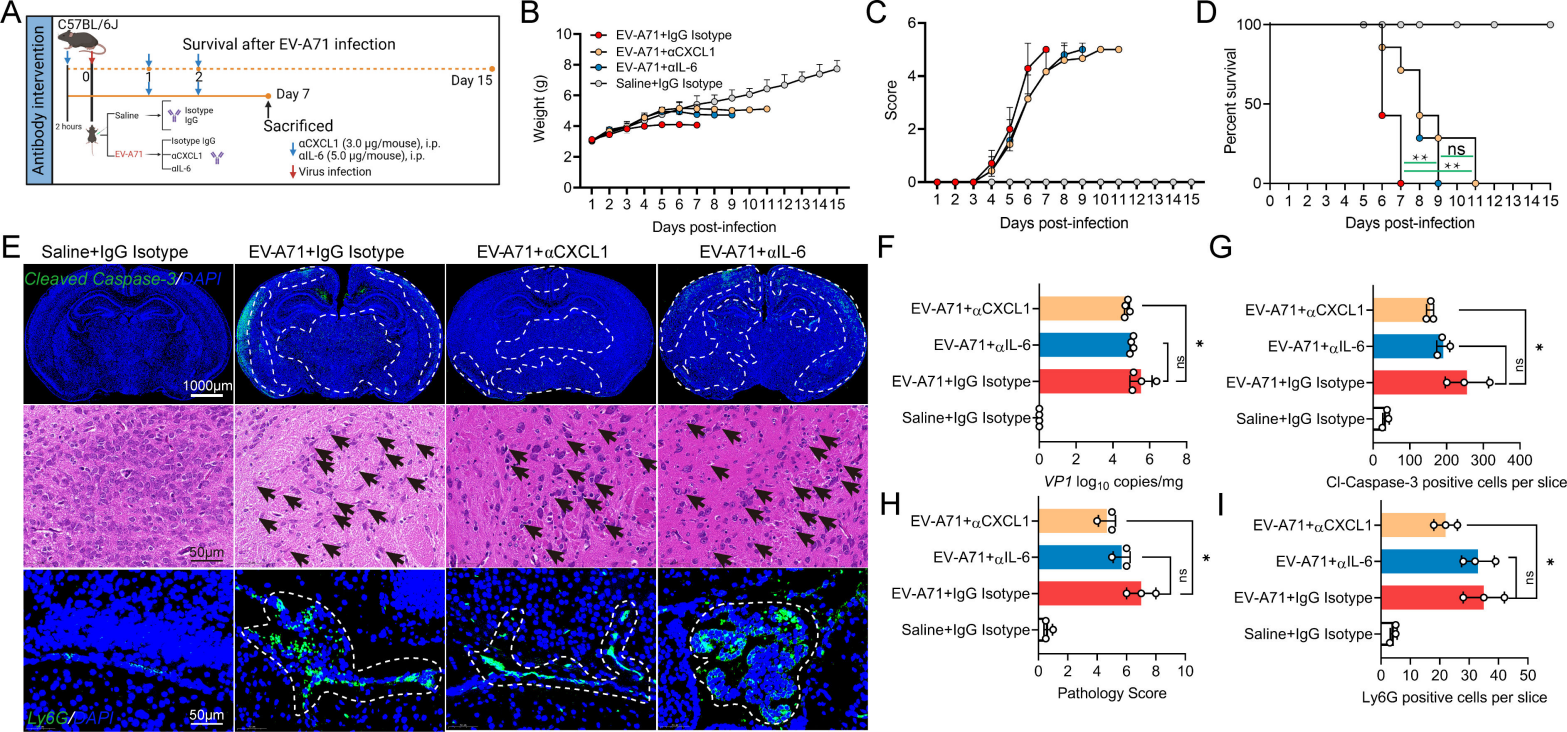
CXCL1 plays a crucial role in EV-A71-induced neuropathogenesis. (**A**) Five-day-old C57/BL6J mice upon EV-A71 infection were treated with αCXCL1 (3.0 µg/mouse) or αIL-6 (5.0 µg/mouse) in the schedule and sacrificed at 7 dpi. The body weight (**B**), clinical scores (**C**), and Kaplan–Meier survival curve (**D**) were recorded from 1 dpi to 15 dpi (*n* = 7). Cleaved Caspase-3, Ly6G immunofluorescence staining, and H&E staining (**E**) were conducted to assess brain pathology (*n* = 3). (**F**) Viral load in brain tissue (*n* = 4). (**G**) Cleaved Caspase-3-positive cells per slice. (**H**) Pathology score. (**I**) Ly6G-positive cells per slice. The white-dotted areas indicate where apoptosis and positive cells mainly occur, and the black arrows indicate neuronal damage and degeneration. *P ＜0.05 vs indicated group and **P ＜0.01 vs indicated group.

We next evaluated the potential therapeutic value of neutralizing CXCL1 in EV-A71 encephalitis. A previous study suggested that astrocyte-derived IL-6 plays an important role in EV-A71 encephalitis (15); therefore, we utilized αCXCL1 and αIL-6 for treatment (Fig. 8A). Compared with αCXCL1- and αIL-6-treated mice with EV-A71 infection, EV-A71-infected mice treated with IgG presented reduced body weights (Fig. 8B) and much higher clinical scores (Fig. 8C). Although antibody therapy did not decrease the mortality rate of EV-A71 infection, it slowed the progression of the disease to some extent, and αCXCL1 therapy was more effective than αIL-6 treatment (Fig. 8D). Furthermore, αCXCL1 treatment significantly ameliorated tissue damage (Fig. 8E) caused by EV-A71, as demonstrated by decreases in the viral load (Fig. 8F), number of Cleaved Caspase-3-positive cells per slice (Fig. 8G), and pathological score (Fig. 8H). Our previous studies revealed neutrophilia in brain tissue after EV-A71 infection (31, 32). CXCL1, a chemokine for neutrophils, may be associated with neutrophilia in brain tissue. As anticipated, the number of Ly6G + neutrophils markedly decreased in EV-A71-infected mice following αCXCL1 administration (Fig. 8E and I). Therefore, our findings suggest that CXCL1 is required for C5a-C5aR1 axis-mediated neuropathogenesis upon EV-A71 infection.

### The p38 MAPK signaling pathway is involved in C5aR1-mediated proinflammatory cytokine production during viral infection

**Fig 9:**
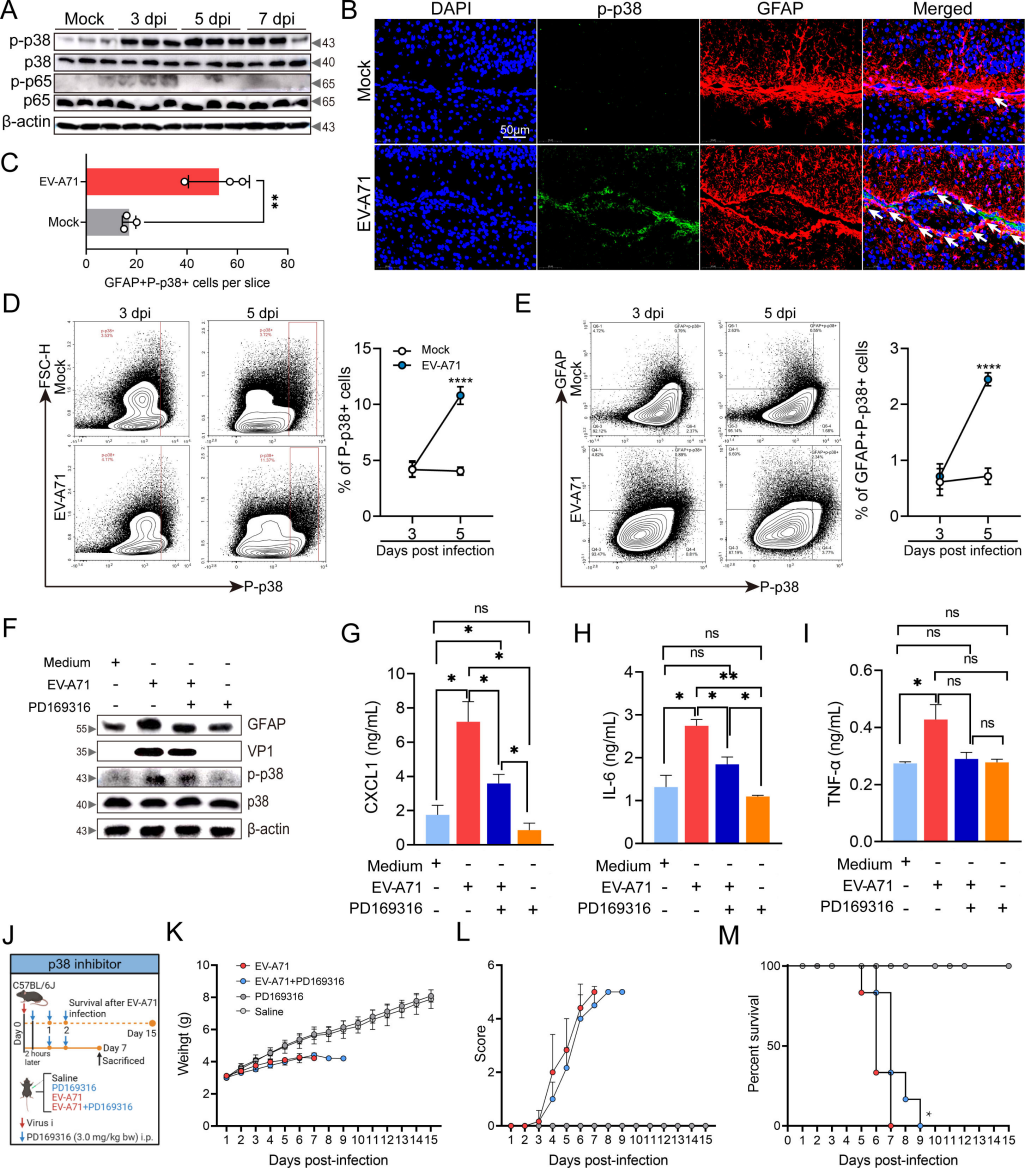
The role of the p38 MAPK signaling pathway in EV-A71-induced neuropathogenesis. Five-day-old C57BL/6J mice were intraperitoneally inoculated with 2.86 × 10^6^ TCID_50_ EV-A71 or saline and sacrificed at 3, 5, and 7 dpi. The phosphorylation of p38 MAPK and NF-κB p65 in the brain tissue was measured by western blot (**A**). The phosphorylation of p38 MAPK in astrocytes of mouse brain was evaluated by immunofluorescence staining (**B**) and FACS (**D, E**) at 3, 5, and 7 dpi (*n* = 5). (**C**) GFAP+ P-p38+ cells per slice. A U87-MG cell population of 2 × 10^6^ was inoculated in a six-well plate, and the virus (MOI = 0.1) and PD169316 (100 nM), was added. Twenty-four hours post-treatment, cell lysates underwent analysis using various antibodies through western blot (**F**). As mentioned above, U87-MG cells were seeded in 96-well plate (1 × 10^4^/well) and were treated with EV-A71 (MOI = 0.1) and PD169316 (100 nM) for different time intervals (*n* = 3). After 24 h, the cell supernatants were harvested for cytokine release assays (**G–I**). (**J**) The schedule of PD169316 administration in 5-day-old C57/BL6J mice upon EV-A71 infection. Five-day-old C57/BL6J mice infected with EV-A71 were given PD169316 at different time points and sacrificed at 7 dpi. The body weight (**K**), clinical scores (**L**), and Kaplan–Meier survival curve (**M**) were recorded from 1 dpi to 15 dpi (*n* = 7). White arrows indicate double-positive cells. The immune blots presented in this figure are derived from three replicates of representative images. *P ＜0.05 vs indicated group, **P ＜0.01 vs indicated group, and ****P ＜0.0001 vs indicated group.

The results described above suggest that the activation of p38 MAPK and NF-κB p65 is regulated by C5aR1. However, while we observed p38 MAPK activation *in vivo*, NF-κB p65 was not significantly activated (Fig. 9A through C). FACS analysis revealed a significant increase in total p38 MAPK phosphorylation in the brain and p38 MAPK phosphorylation in astrocytes at 5 days following EV-A71 infection compared with the control group of mice (Fig. 9D and E). To gain insight into whether CXCL1 production is associated with p38 activation, we used PD169316, a selective p38 MAPK phosphorylation inhibitor, for subsequent experiments. As shown in Fig. 9F through I, PD169316 treatment inhibited the phosphorylation of p38 MAPK, astrocyte activation, and VP1 expression. In addition, the EV-A71-induced production of CXCL1 and IL-6 significantly decreased in U87-MG cells after PD169316 treatment. We next confirmed the potential therapeutic value of PD169316 for EV-A71-induced neuropathological changes. As shown in Fig. 9J through M, although PD169316 administration did not decrease the mortality rate of EV-A71 infection, it slowed the progression of the disease to some extent. Taken together, our findings highlight that the p38 MAPK signaling pathway is important for C5aR1-mediated proinflammatory cytokine production during viral infection. The treatment with PD169316 demonstrated a degree of efficacy in slowing the progression of the disease; however, it did not reverse the mortality caused by EV-A71.

## DISCUSSION

Astrocytes, which are abundant glial cells, have emerged as pivotal regulators of neuroinflammation (13). Our previous study demonstrated that EV-A71 preferentially infects astrocytes and results in astrogliosis (14, 33). Similarly, a previous study revealed that EV-A71 mainly replicates in astrocytes in the mouse brain (15). GFAP levels in human plasma can be used to accurately identify astrogliosis in the CNS (34). Therefore, we first determined where EV-A71 replicates in the mouse brain. Our immunofluorescence analyses revealed that EV-A71 VP1 was localized mainly in astrocytes (GFAP+), as opposed to neurons (NeuN+) or microglia (IBA1+). Our case–control study revealed an increase in plasma GFAP levels in patients with severe EV-A71 infection, suggesting that the activation of astrocytes is associated with the occurrence of EV-A71 encephalitis. Our findings revealed that EV-A71 could effectively replicate in astrocytes, triggering intracellular signaling pathways (the p38 MAPK and NF-κB pathways) and the subsequent release of proinflammatory cytokines. Extensive evidence supports the notion that the transcription factors p38 MAPK and NF-κB regulate various facets of the proinflammatory response in astrocytes (13). Many studies have suggested that EV-A71 can result in human astrocytoma (U251) cell autophagy and apoptosis (35, 36). However, there is limited evidence on the mechanism of astrocyte activation upon EV-A71 infection.

The C5a–C5aR1 axis performs fundamental functions in the modulation of neuroinflammation (19, 37) and neurodegeneration (21, 38). Most neural cells (e.g., astrocytes) are capable of synthesizing a complete and functional complement system (39). To explore the role of complement components in EV-A71-induced astrocyte activation, we examined the expression of several complement molecules. Our findings revealed that EV-A71 activated the complement C5a–C5aR1 axis in astrocytes and that C5aR1 knockdown or blockade reduced astrocyte activation and proinflammatory cytokine production. Another study (37) revealed that C5aR1 deficiency decreased astrocyte activation induced by spinal cord injury, indicating the impact of C5aR1 on astrocyte function. Notably, our observations demonstrated that EV-A71 infection led to the activation of the C5a–C5aR1 axis in astrocytes and severe neuroinflammation in the mouse brain. Similarly, activation of the C5a–C5aR1 axis has been detected in other models of viral encephalitis, such as models of Zika virus (40), Middle East Respiratory Syndrome Coronavirus (41), and HIV-1 infection (42). Therefore, we speculated that the C5a–C5aR1 axis may contribute to neuroinflammation upon EV-A71 infection. We used a mouse model of EV-A71 infection induced by intraperitoneal injection suitable for assessing EV-A71 neurovirulence (23, 33). Further experiments revealed a reduction in EV-A71-induced neuroinflammation and tissue damage in C5aR1^−/−^ mice and PMX53-treated mice. Our results also revealed that in patients with encephalitis, the complement cascade, especially the C5a–C5aR1 axis, was significantly activated in the plasma. Abnormal complement activation triggers self-attack, and overreaction of the body is the cause of many acute infectious diseases (43, 44). A growing body of evidence has demonstrated complement overactivation in sepsis (44), viral hepatitis (45), human infections with highly pathogenic avian influenza viruses H5N1/H7N9 (46, 47), and C5a inhibitor or C5aR1 knockout is effective in alleviating symptoms of these diseases and reducing infection-induced lethality *in vivo*. Hence, the complement system plays a pivotal role in safeguarding against microbial invasion, but its aberrant activation also serves as a potent driver of inflammatory diseases.

Astrocytes secrete the proinflammatory cytokines CXCL1, IL-6, and TNF-α, which contribute to CNS inflammation (13). Our findings confirmed that the C5a–C5aR1 axis regulates the EV-A71-induced production of proinflammatory cytokines, especially CXCL1. Our observations revealed that CXCL1 in the brain was predominantly derived from astrocytes upon EV-A71 infection and a substantial positive correlation between the expression of CXCL1 and the activation of astrocytes in EV-A71 infection patients was observed. These findings underscore the crucial role of astrocyte-derived CXCL1 in the potentiation of inflammatory cascades in the CNS. Similar observations have been reported in HSV-1 infection (30) and experimental autoimmune encephalomyelitis (48). It has been reported that astrocyte-derived IL-6 is associated with neuropathological changes upon EV-A71 infection (15). We next utilized neutralizing antibodies to evaluate the roles of CXCL1 and IL-6 in EV-A71 encephalitis. Compared with IgG treatment, αCXCL1 or αIL-6 administration slowed the progression of the disease, although these treatments were unable to decrease the mortality rate of EV-A71 infection. On the basis of our observations, it seemed that αCXCL1 administration exerted a stronger therapeutic effect than αIL-6. Our previous studies (14, 49–51) revealed the involvement of neutrophilia and neutrophil activation in encephalitis due to enteroviral infection. The critical function of CXCL1 involves promoting the recruitment of neutrophils to the site of injury (13). Our results showed that neutralizing CXCL1 significantly inhibited neutrophil migration into the brain. Neutrophils are rarely observed in the brain owing to the highly selective BBB. Nevertheless, inflammatory conditions disrupt the BBB and cause neuroinflammation (52). Therefore, we speculate that astrocyte-derived CXCL1 drives neutrophil transmigration, and future experiments should be performed to elucidate the mechanisms underlying neutrophil-related alterations in EV-A71 encephalitis.

As mentioned above, the p38 MAPK and NF-κB signaling pathways are activated in astrocytes. Through western blot analysis, we found significant activation of only p38 MAPK in mouse brain tissue after EV-A71 infection. We subsequently detected p38 activation in astrocytes via immunofluorescence staining. The regulation of EV-A71 neurovirulence is associated with the p38 MAPK signaling pathway (53). Our *in vitro* experiments further revealed that blockade of p38 MAPK reduced astrocyte activation and proinflammatory cytokines (e.g., CXCL1 and IL-6) production. In addition, pharmacological blockade of p38 activation by PD169316 slowed the progression of EV-A71 infection. Similar observations have been reported for SFTSV, HSV-1, and SARS-CoV-2 infection (54). Taken together, our findings suggest the involvement of the p38 MAPK signaling pathway in C5aR1-mediated neuropathological changes during EV-A71 infection.

### Conclusion

**Fig 10:**
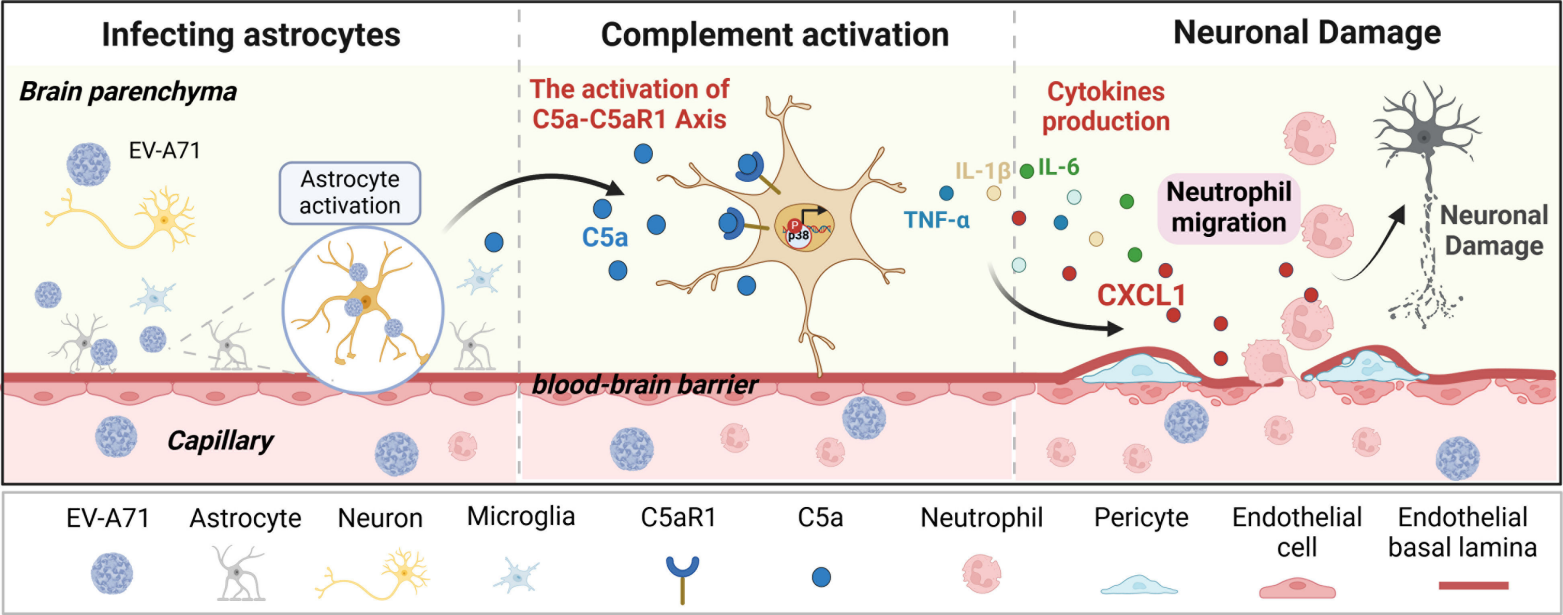
A proposed mechanism underlying the regulation of neuropathogenesis upon EV-A71 infection. EV-A71 preferentially infects and replicates in astrocytes of mice and triggers the C5a–C5aR1 signaling, which in turn dominantly promote CXCL1 and IL-6 production in astrocytes. The production of CXCL1 promoting the recruitment of neutrophils into the injured site. All events together disrupt brain homeostasis and finally lead to neuroinflammation and tissue damage.

Overall, our work demonstrates that activation of the complement C5a–C5aR1 axis in astrocytes facilitates the neuropathological changes caused by EV-A71 infection, emphasizing the pivotal role of p38 MAPK-mediated CXCL1 production in these alterations (Fig. 10). Thus, inhibiting the C5a–C5aR1 axis has emerged as a potential therapeutic strategy to mitigate CNS injury induced by EV-A71 infection.

## Data Availability

All data are presented in the results or supplemental material.

## SUPPLEMENTAL MATERIAL

Figure S1 jvi.01514-24-s0001.docx Expression of C5aR1 in astrocytes.

Figure S2 jvi.01514-24-s0002.docx Cytokine expression in nerve cells from EV-A71-infected brains.

Figure S3 jvi.01514-24-s0003.docx Quantified western blots.

Table S1 jvi.01514-24-s0004.doc Demographic information of the participants.

Table S2 jvi.01514-24-s0005.doc Human primer sequences.

Table S3 jvi.01514-24-s0006.doc Mouse primer sequences.

ASM does not own the copyrights to Supplemental Material that may be linked to, or accessed through, an article. The authors have granted ASM a non-exclusive, world-wide license to publish the Supplemental Material files. Please contact the corresponding author directly for reuse.
