# Supplementary material for: The activation of complement C5a-C5aR1 axis in astrocytes facilitates the neuropathogenesis due to EV-A71 infection by upregulating CXCL1
Source: J Virol. 2024 Dec 16;99(1):e01514-24. doi: 10.1128/jvi.01514-24 (PMC11784463; doi:10.1128/jvi.01514-24)
Supplement: Figure S1 — Expression of C5aR1 in astrocytes. [file jvi.01514-24-s0001.docx]

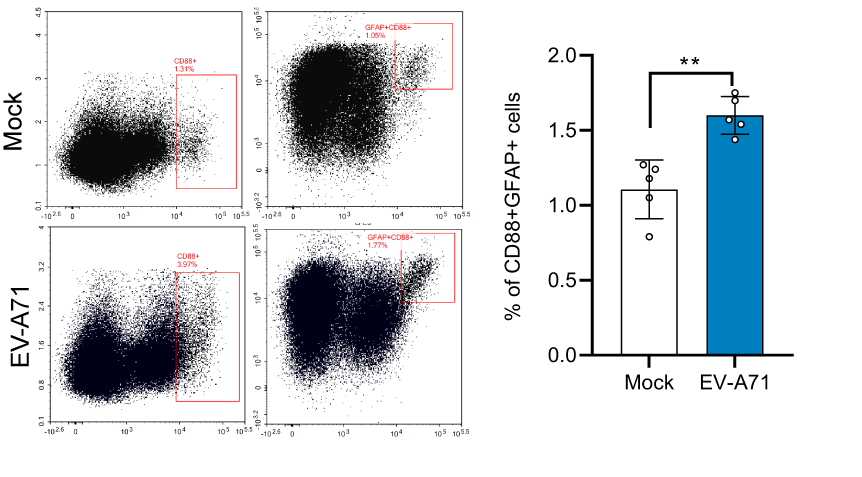
**Supplementary Figure 1. EV-A71 infection promotes the expression of C5aR1 on astrocytes in mouse brain.** Five-day-old C57BL/6J mice were intraperitoneally inoculated with 2.86×10^6^ TCID_50_ EV-A71 or saline, and sacrificed at 5 dpi. The expression of C5aR1 on astrocytes in mouse brain was evaluated by FACS (n=5),***P* ＜0.01, vs Mock.
