# Supplementary material for: The activation of complement C5a-C5aR1 axis in astrocytes facilitates the neuropathogenesis due to EV-A71 infection by upregulating CXCL1
Source: J Virol. 2024 Dec 16;99(1):e01514-24. doi: 10.1128/jvi.01514-24 (PMC11784463; doi:10.1128/jvi.01514-24)
Supplement: Figure S2 — Cytokine expression in nerve cells from EV-A71-infected brains. [file jvi.01514-24-s0002.docx]

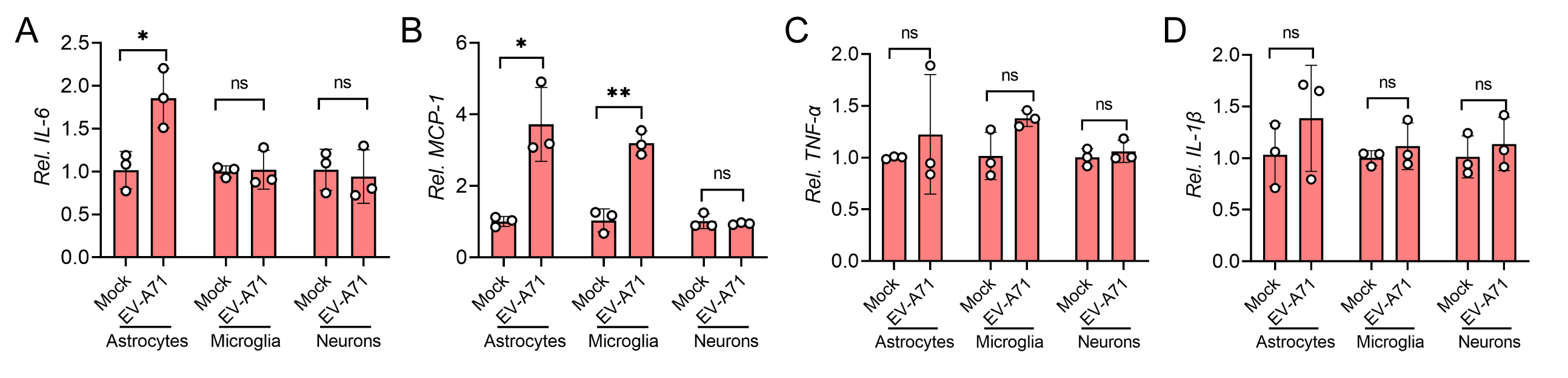


**Supplementary Figure 2. Cytokines expression in nerve cells from EV-A71-infected brains.** Five-day-old C57BL/6J mice were intraperitoneally inoculated with 2.86×10^6^ TCID_50_ EV-A71 or saline, and sacrificed at 7 dpi. The mRNA expression of IL-6 **(A)**, MCP-1 **(B)**, TNF-α **(C)**, and IL-1β **(D)** in primary astrocytes, microglia, and neurons isolated from EV-A71-infected mice was detected by qRT-PCR (n=3). **P* ＜0.05, vs Mock; ***P* ＜0.01, vs Mock.
