# Supplementary figures and images for: The activation of complement C5a-C5aR1 axis in astrocytes facilitates the neuropathogenesis due to EV-A71 infection by upregulating CXCL1
Source: J Virol. 2024 Dec 16;99(1):e01514-24. doi: 10.1128/jvi.01514-24 (PMC11784463; doi:10.1128/jvi.01514-24)

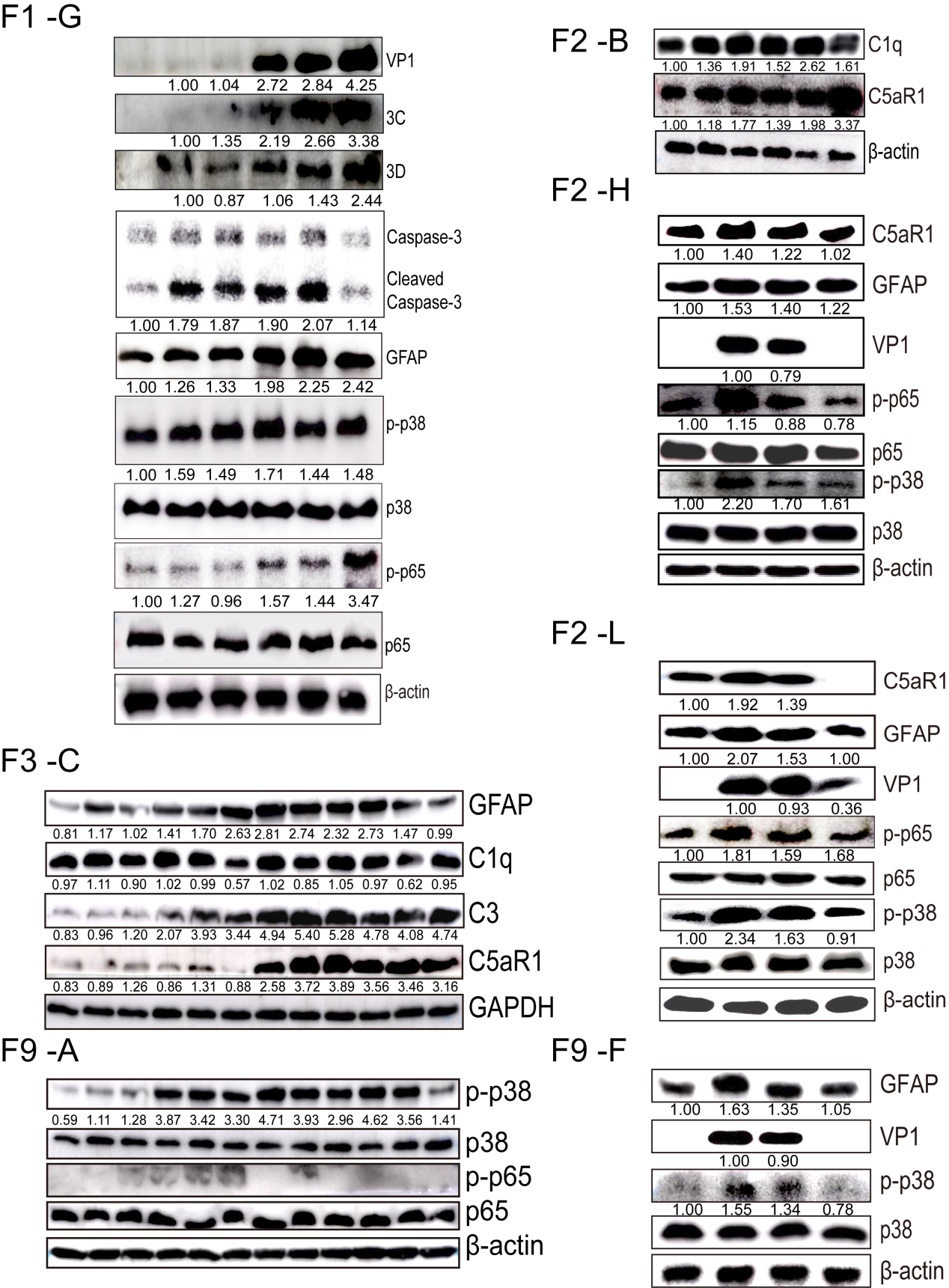

Supplement: Figure S3 — Quantified western blots. [file jvi.01514-24-s0003.docx]
