# Supplementary material for: The activation of complement C5a-C5aR1 axis in astrocytes facilitates the neuropathogenesis due to EV-A71 infection by upregulating CXCL1
Source: J Virol. 2024 Dec 16;99(1):e01514-24. doi: 10.1128/jvi.01514-24 (PMC11784463; doi:10.1128/jvi.01514-24)
Supplement: Table S1 — Demographic information of the participants. [file jvi.01514-24-s0004.doc]

**Supplementary Table 1**

**The demographic information and clinical laboratory results of the participants**

| **Variables** | **Mild**  **(n=75)** | **Severe**  **(n=75)** | ***χ2/z/t*** | ***P*** |
| --- | --- | --- | --- | --- |
| Gender (male) n, % | 49(65.33%) | 47(62.66%) | 0.116 | 0.865 |
| Age (month) | 22(15,36) | 19(13,30) | -1.085 | 0.278 |
| WBC (10^9^/L) | 10.158±3.26973 | 11.4111±4.64435 | 0.626 | 0.532 |
| RBC (10^12^/L) | 4.4686±0.3578 | 4.4323±0.35189 | 2.467 | 0.015 |
| HGB (g/L) | 119.16±11.50563 | 114.68±10.72527 | 0.638 | 0.524 |
| HCT (%) | 33.732±2.52459 | 33.4493±2.88661 | -2.812 | 0.006 |
| Platelets (10^9^/L) | 292.88±88.86263 | 334.16±90.92335 | -0.012 | 0.991 |
| MCV (fL) | 76.45(74.15,78.7) | 76.4(72.6,78.7) | -0.554 | 0.579 |
| MCH (pg) | 26.65(25.8,28.6) | 26.3(24.5,27.3) | -2.013 | 0.044 |
| MCHC (g/L) | 350.5(344.0,364.0) | 342.0(326.0,359.0) | -2.502 | 0.012 |
| MPV (fL) | 9.7(9.1,10.3) | 9.6(9.1,10.1) | -0.472 | 0.637 |
| PCT% | 0.275(0.23,0.235) | 0.31(0.26,0.38) | -3.115 | 0.002 |
| Neutrophil ratio (%) | 51.0027±21.54681 | 47.6242±23.12065 | -0.909 | 0.365 |
| Lymphocyte ratio (%) | 40.8947±20.06048 | 43.9983±21.71666 | 2.082 | 0.039 |
| Monocyte ratio (%) | 6.3809±2.04107 | 5.698±1.97608 | -1.866 | 0.064 |
| Eosinophil ratio (%) | 1.3856±1.56341 | 1.9458±2.01221 | 1.772 | 0.079 |
| Basophil ratio (%) | 0.3046±0.33957 | 0.2208±0.21026 | -0.988 | 0.325 |
| Neutrophil count (10^9^/L) | 5.3341±3.32937 | 6.0197±5.0013 | -1.765 | 0.080 |
| Lymphocyte count (10^9^/L) | 3.879±1.92798 | 4.4738±2.1906 | 1.085 | 0.280 |
| Monocyte count (10^9^/L) | 0.7208±0.85384 | 0.6084±0.27511 | -1.481 | 0.141 |
| Eosinophil count (10^9^/L) | 0.1311±0.15374 | 0.1705±0.17219 | 1.797 | 0.075 |
| Basophil count (10^9^/L) | 0.0307±0.03422 | 0.0225±0.01911 | -1.453 | 0.148 |
| RDWSD (%) | 36.8165±4.87682 | 38.0595±5.51292 | -0.613 | 0.541 |
| RDWCV (%) | 14.0892±3.2329 | 14.356±1.92039 | -0.636 | 0.526 |
| PDW (%) | 10.7068±1.43759 | 10.988±3.52401 | 0.439 | 0.661 |
| CRP (mg/L) | 12.2425±15.70432 | 11.1034±15.9508 | 4.916 | ＜0.001 |
| TBIL (μmol/L) | 11.28±2.984 | 8.73±3.359 | 2.133 | 0.035 |
| DBIL (μmol/L) | 3.0(2.0,3.0) | 2.0(2.0,3.0) | -3.355 | ＜0.001 |
| IBIL (μmol/L) | 8.54±2.844 | 6.39±2.953 | -6.107 | ＜0.001 |
| ALT (U/L) | 15.73±9.193 | 26.6±12.376 | 1.873 | 0.063 |
| AST (U/L) | 43.92±16.88095 | 39.4514±11.74985 | 0.82 | 0.414 |
| ALP (U/L) | 237.69±104.832 | 217.35±187.742 | 0.933 | 0.352 |
| GGT (U/L) | 14.7824±36.17353 | 10.8446±2.98389 | 0.649 | 0.517 |
| Total protein (g/L) | 69.83±4.275 | 69.33±5.234 | 0.732 | 0.465 |
| Albumin (g/L) | 42.35±3.207 | 41.95±3.433 | 0.205 | 0.838 |
| Globulin (g/L) | 27.49±2.551 | 27.38±3.845 | -1.105 | 0.271 |
| LDH (U/L) | 241.9311±49.95463 | 252.8479±68.49564 | -1.673 | 0.097 |
| CK (U/L) | 88.47±45.587 | 111.18±108.408 | 0.944 | 0.347 |
| CK-MB (U/L) | 21.76±10.199 | 20.17±10.397 | 0.512 | 0.609 |
| Urea (mmol/L) | 3.31±0.827 | 3.23±0.984 | 1.340 | 0.183 |
| Creatinine (μmol/L) | 28.4±4.568 | 27.28±5.618 | 0.597 | 0.551 |
| Uric acid (μmol/L) | 260.0667±72.68171 | 252.2108±87.39396 | -2.251 | 0.026 |
| K (mmol/L) | 3.91±0.376 | 4.09±0.584 | -2.895 | 0.004 |
| Na (mmol/L) | 134.77±3.494 | 136.33±3.089 | 0.134 | 0.893 |
| Cl (mmol/L) | 100.63±3.448 | 100.56±2.84 | -3.121 | 0.002 |
| Ca (mmol/L) | 2.4099±0.0962 | 2.4707±0.13862 | 0.405 | 0.686 |

WBC: white blood cells; RBC: red blood cells; HGB: hemoglobin; HCT: hematocrit; MCV: mean red blood cell volume; MCH: mean content of hemoglobin; MCHC: mean hemoglobin concentration; MPV: average platelet volume; PCT: Platelet hematocrit; RDWSD: red blood cell distribution width; RDWCV: red blood cell distribution width; PDW: Platelet distribution width; CRP: C-reactive protein; TBIL: total bilirubin; DBIL: direct bilirubin; IBIL: indirect bilirubin; ALT: alanine aminotransferase; AST: aspartate aminotransferase; ALP: alkaline phosphatase; GGT: glutaminyltransferase; LDH: lactate dehydrogenase; CK: creatine kinase; CK-MB: creatine kinase isoenzymes. There were 18 children in the control group, including 10 males, with an average age of 42 months.
