# Supplementary material for: The activation of complement C5a-C5aR1 axis in astrocytes facilitates the neuropathogenesis due to EV-A71 infection by upregulating CXCL1
Source: J Virol. 2024 Dec 16;99(1):e01514-24. doi: 10.1128/jvi.01514-24 (PMC11784463; doi:10.1128/jvi.01514-24)
Supplement: Table S2 — Human primer sequences. [file jvi.01514-24-s0005.doc]

**Supplementary Table 2**

**Human** Primer sequences

| **Gene** | **Forward** | **Reverse** | **Product lengths** |
| --- | --- | --- | --- |
| CFB | CGTGTGTCCTTCTGGCTTCT | CGAAGTCGTGTGGTCTTGGA | 149 |
| GBP2 | AGGAGGAAGAGCTGAACCCT | CGAGGCCCATTGACTGGAAT | 115 |
| FBLN5 | GTTCCCGCTGACATCTTCCA | GCCTCATATGCAGTGGTGCT | 134 |
| SERPING | ATTCTCCTACCCAGCCCACT | ACACGGCCTCTGTTGAATGA | 90 |
| C5aR1 | GCTTCCTGTGGCCTCTACTC | TTGAGTGTCTTGGTGGACCG | 97 |
| GAPDH | CAGGAGGCATTGCTGATGAT | GAAGGCTGGGGCTCATTT | 138 |
