# Supplementary material for: The activation of complement C5a-C5aR1 axis in astrocytes facilitates the neuropathogenesis due to EV-A71 infection by upregulating CXCL1
Source: J Virol. 2024 Dec 16;99(1):e01514-24. doi: 10.1128/jvi.01514-24 (PMC11784463; doi:10.1128/jvi.01514-24)
Supplement: Table S3 — Mouse primer sequences. [file jvi.01514-24-s0006.doc]

**Supplementary Table 3**

**Mouse** Primer sequences

| Gene | Forward | Reverse | Product lengths |
| --- | --- | --- | --- |
| IL-6 | CTGCAAGAGACTTCCATCCAG | AGTGGTATAGACAGGTCTGTTGG | 131 |
| TNF-α | CCTGTAGCCCACGTCGTAG | GGGAGTAGACAAGGTACAACCC | 148 |
| CXCL1 | GCCACACTCAAGAATGGTCG | ACTTGGGGACACCTTTTAGCAT | 94 |
| IL-1β | GAAATGCCACCTTTTGACAGTG | TGGATGCTCTCATCAGGACAG | 116 |
| MCP-1 | TAAAAACCTGGATCGGAACCAAA | GCATTAGCTTCAGATTTACGGGT | 120 |
| C5aR1 | TTTGATGCCACCGCCTGTAT | AAACGGTCGGCACTAATGGT | 95 |
| C1q | ACTGAAGGGCGTGAAAGGCAATC | TTCTGGTATGGACTCTCCTGGTTGG | 144 |
| C5a | AAACCTGTGAGGAGCGAGTG | TTGGACAGGTTTATGGGGGC | 119 |
| C3aR1 | TAGCCTGGACCGATGTCTGA | CGGGCACACACATCACAAAG | 122 |
| C3 | GTTCGGCATAGAGAAGAGGCAAGAG | TTGTTGAAGGCAGCATAGGCAGAG | 105 |
| β-actin | GTGCTATGTTGCTCTAGACTTCG | ATGCCACAGGATTCCATACC | 174 |
